# Genetic Loci and Novel Discrimination Measures Associated with Blood Pressure Variation in African Americans Living in Tallahassee

**DOI:** 10.1371/journal.pone.0167700

**Published:** 2016-12-21

**Authors:** Jacklyn Quinlan, Laurel N. Pearson, Christopher J. Clukay, Miaisha M. Mitchell, Qasimah Boston, Clarence C. Gravlee, Connie J. Mulligan

**Affiliations:** 1 Department of Anthropology, University of Florida, Gainesville, FL, United States of America; 2 Genetics Institute, University of Florida, Gainesville, FL, United States of America; 3 Department of Anthropology, Pennsylvania State University, University Park, PA, United States of America; 4 Health Equity Alliance of Tallahassee (HEAT), Tallahassee, FL, United States of America; 5 Greater Frenchtown Revitalization Council, Tallahassee, FL, United States of America; 6 Florida Department of Children and Families Substance Abuse and Mental Health Program, Tallahassee, FL, United States of America; Oklahoma Medical Research Foundation, UNITED STATES

## Abstract

Sequencing of the human genome and decades of genetic association and linkage studies have dramatically improved our understanding of the etiology of many diseases. However, the multiple causes of complex diseases are still not well understood, in part because genetic and sociocultural risk factors are not typically investigated concurrently. Hypertension is a leading risk factor for cardiovascular disease and afflicts more African Americans than any other racially defined group in the US. Few genetic loci for hypertension have been replicated across populations, which may reflect population-specific differences in genetic variants and/or inattention to relevant sociocultural factors. Discrimination is a salient sociocultural risk factor for poor health and has been associated with hypertension. Here we use a biocultural approach to study blood pressure (BP) variation in African Americans living in Tallahassee, Florida by genotyping over 30,000 single nucleotide polymorphisms (SNPs) and capturing experiences of discrimination using novel measures of unfair treatment of self and others (n = 157). We perform a joint admixture and genetic association analysis for BP that prioritizes regions of the genome with African ancestry. We only report significant SNPs that were confirmed through our simulation analyses, which were performed to determine the false positive rate. We identify eight significant SNPs in five genes that were previously associated with cardiovascular diseases. When we include measures of unfair treatment and test for interactions between SNPs and unfair treatment, we identify a new class of genes involved in multiple phenotypes including psychosocial distress and mood disorders. Our results suggest that inclusion of culturally relevant stress measures, like unfair treatment in African Americans, may reveal new genes and biological pathways relevant to the etiology of hypertension, and may also improve our understanding of the complexity of gene-environment interactions that underlie complex diseases.

## Introduction

African Americans bear a disproportionate burden of ill health in the United States (US) [1] with cardiovascular disease being the leading contributor to reduced life expectancy. Hypertension is a leading risk factor of cardiovascular disease and afflicts more African Americans than any other racially defined group in the US [2]. The high prevalence and premature onset of hypertension in African Americans is a major public health concern [3].

The high estimated heritability of blood pressure (BP) has [4] prompted extensive efforts to identify the genetic underpinnings of BP variation [5]. However, traditional genetic methods such as genome-wide association studies (GWASs) and admixture mapping have largely failed to identify replicable loci that associate with BP across populations [6–10]. Out of the two GWASs for BP performed in African Americans [8, 9], no replicable loci reached genome-wide significance. Some studies identified a positive relationship between African ancestry and BP [11, 12], while others failed to find any significant relationship [13, 14]. The lack of replication of genetic loci associated with BP across populations of different ancestries has generally been attributed to population-specific genetic variants, variation in allele frequencies, different patterns of linkage disequilibrium (LD) across populations or low statistical power due to limited sample size, particularly in African-Americans [15].

While it is true that population-specific variants, allele frequencies, and LD exist, it’s not clear that these differences account for the lack of replication across studies. In fact, epidemiological evidence suggests that environmental factors contribute more to phenotypic variation in BP than do genetics [16, 17]. BP levels vary widely across populations, with the prevalence of hypertension lowest in populations with low levels of environmental and psychological stress and extensive genetic variability [18], suggesting that the major determinants of high BP are likely to be a constellation of sociocultural factors, with genetic determination being limited to variation within populations and to interactions with the environment [18].

Sociocultural factors [19–24] are important determinants of hypertension, and discrimination is a salient risk factor for poor health [25]. Some researchers propose that discrimination may contribute to the health disparities observed in African Americans [19] since discrimination has been associated with many health issues, including BP in African Americans [26–28]. Most existing studies measure perceived discrimination only by reference to participants' personal exposure to unfair treatment. However, we have shown that unfair treatment experienced by individuals close to the study participant—family or friends—is an important stressor [29].

Multiple studies have discussed the “missing heritability” of complex phenotypes, i.e. the discrepancy between the estimated heritability of a phenotype and the total variation explained by specific genetic variants [30–32]. The missing heritability of complex disease may partially be due to the lack of inclusion of environmental stressors in standard genetic association studies [33]. The definition of heritability is often equated with the genetic contribution to phenotypic variation, but it actually refers to the ratio of genetic to total phenotypic variance in a population [34]. Phenotypic variance includes genetic and environmental variation as well as a covariance term between the two, but many genetic studies assume this covariance term to be zero. If the covariance is not zero or if there are gene-environment interactions, then standard genetic association studies that do not include relevant environmental factors may miss important associations. With a few exceptions [20, 29, 35], research into the stressors associated with BP seldom includes both genetic and sociocultural data, such that few studies are able to evaluate interactions.

Here we conduct a biocultural investigation of BP among 157 African Americans living in Tallahassee, Florida (USA). We use a Bayesian joint admixture and association method that prioritizes regions of the genome with African ancestry [36] and improves on other methods [36–38] by taking advantage of the reduced testing burden of admixture mapping relative to association mapping. We adapt this method to include both genetic and sociocultural data, and interaction effects, to test for association with BP. We performed simulation analyses to assess the statistical significance of our associations. We assay over 30,000 genetic markers [ancestry informative markers (AIMs) and SNPs in genes associated with hypertension, cardiovascular disease, and stress] and include new measures of unfair treatment in order to develop an expanded picture of risk factors underlying BP variation.

## Results

### Study sample characteristics

Sample characteristics for the 157 African American participants are detailed in Table 1. There were statistically significant differences in sex, age, BMI, SBP, and DBP readings between those individuals taking and not taking antihypertensive medications.

**Table 1:** Sample characteristics.

Characteristics	Taking BP meds	Not taking BP meds	Total sample
N	48	109	157
Men: Women	9:39	41:68*	50:107
Age (years) (SD)	47.48 (10.2)	38.24 (11.6)*	41.06 (11.9)
Body Mass Index (SD)	38.67 (10.0)	29.67 (7.7)*	32.36 (9.4)
Systolic BP (SD)	136.88 (20.9)	124.58 (19.9)*	128.34 (20.9)
†Systolic BP_adj10_ (SD)	146.88 (20.9)	124.58 (19.9)*	131.40 (22.7)
Diastolic BP (SD)	85.33 (12.0)	79.61 (13.5)*	81.36 (13.3)
†Diastolic BP_adj5_ (SD)	90.33 (12.0)	79.61 (13.5)*	82.89 (13.9)
Education (years) (SD)	13.13 (2.5)	13.16 (2.4)	13.15 (2.4)
Global African Ancestry (SD)	0.79 (0.06)	0.79 (0.05)	0.79 (0.05)
**UT-Self (range)	1.67 (0–8)	1.60 (0–9)	1.62 (0–9)
**UT-Other (range)	1.65 (0–6)	1.41 (0–7)	1.48 (0–7)
**UT-Self dichotomous (yes) (%)	27 (27.6%)	71 (71.4%)	98 (62.4%)
**UT-Other dichotomous (yes) (%)	37 (34.6%)	70 (65.4%)	107 (68.2%)

†Antihypertensive medication use was accounted for by adding 10mmHg and 5mmHg to SBP and DBP, respectively.

*Significant difference between individuals taking and not taking antihypertensive medications (p<0.01).

**UT indicates a measure of unfair treatment.

### Variable selection for inclusion in BP models

In our sample, age, sex, and BMI were associated with SBP (S1 Table). Unfair treatment variables were not associated with BP in simple linear regression analysis. Based on these results, as well as potential confounders previously identified in the literature [39] and inclusion of our new measures of unfair treatment, we included the following variables in our models of adjusted BP: age, sex, BMI, education, African ancestry, unfair treatment and PC-AiRs.

### Strategy for genetic analyses

**Fig 1:**
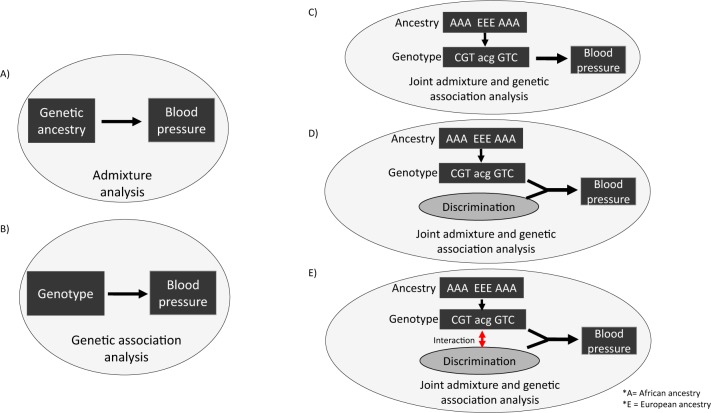
Illustration of the analyses performed. A) **S**tandard admixture mapping using a frequentist approach tested for association between genetic ancestry and BP. B) Standard association mapping using a frequentist approach tested for association between SNP and BP. Three progressive Bayesian joint admixture and genetic association analyses for BP were performed that prioritized regions of the genome with African ancestry when evaluating the strength of the association between a SNP and BP. C) Model 1 tested for association between SNP genotype and BP, D) Model 2 included discrimination measures, E) Model 3 tested for interaction effects between SNPs and discrimination measures that are associated with BP

We analyzed SBP and DBP measurements separately since the underlying genetic susceptibility of hypertension may be different for elevated SBP and DBP. Three main genetic analyses were performed (Fig 1). We first performed standard admixture mapping using a frequentist approach by linearly regressing blood pressure on local ancestry, adjusted for global ancestry, sex, age, education, BMI and relatedness (Fig 1A). We next performed standard genetic association analysis for blood pressure using a frequentist approach by linearly regressing blood pressure on SNP genotype, adjusted for global ancestry, sex, age, education, BMI, and relatedness (Fig 1B). Finally, we used joint admixture and association mapping with partial Bonferroni correction [36] to identify SNPs that were associated with BP using a Bayesian approach where we set the posterior probabilities from admixture mapping as the prior probabilities for association mapping. Three progressive joint admixture and association models were performed. Model 1 tested SNP genotype (Fig 1C), Model 2 added measures of unfair treatment (Fig 1D), and Model 3 tested for an interaction term between SNP genotype and unfair treatment (Fig 1E).

### Admixture mapping for blood pressure

Admixture mapping (illustrated in Fig 1A) did not detect any genome-wide significant loci associated with BP after correcting for the average number of admixture switches (p-value less than or equal to 0.05/78.82 = 6.34x10^-4^) (S1A and S1B Fig).

### Association mapping for blood pressure

Association mapping (illustrated in Fig 1B) did not detect any significant SNPs associated with BP after Bonferroni correction (p-value less than or equal to 1/27,559 = 3.6 x 10^−5^) (S1C and S1D Fig).

### Joint admixture and association mapping for BP

Three progressive joint admixture and association models were performed using a Bayesian approach (illustrated in Fig 1C–1E); Model 1 tested SNP genotype, Model 2 added measures of unfair treatment, and Model 3 tested for an interaction term between SNP genotype and unfair treatment. We performed simulation analyses to assess the statistical significance of our associations. We randomly re-shuffled the associated BP measures and covariates to the SNP genotypes and ancestry estimates in our participants 10,000 times and created a distribution of p-values. This distribution was used to obtain the false positive rate of our associations. We only report significant SNPs that were confirmed through simulations analyses.

**Fig 2:**
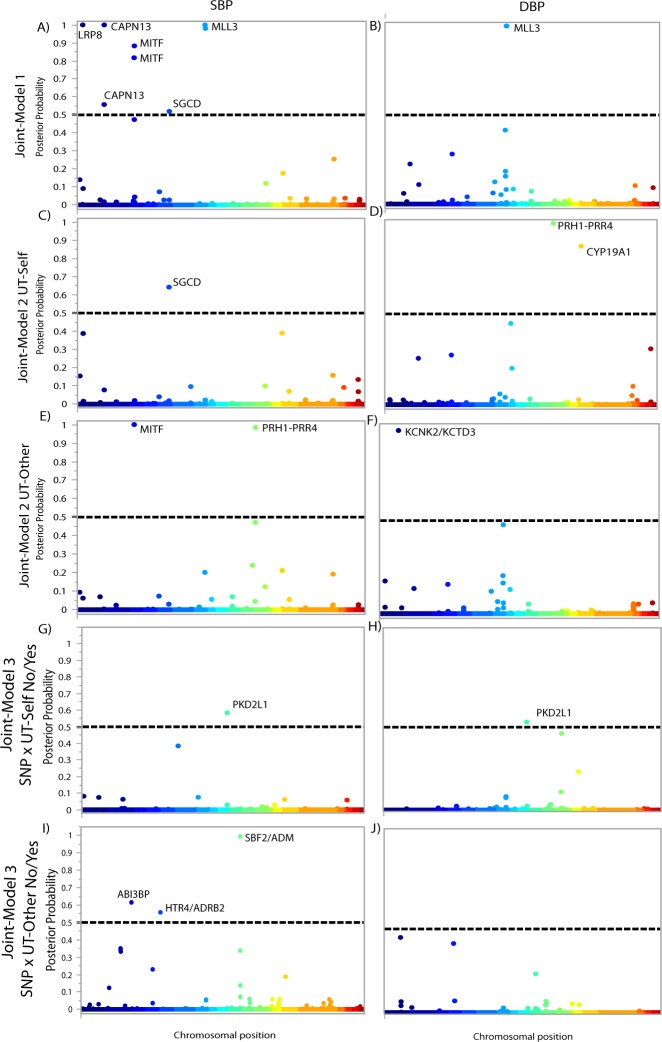
Bayesian Manhattan plots for joint ancestry and association testing with BP. Each association is plotted based on its chromosomal position (x axis) and the posterior probability that a locus affects BP (y axis). The dashed line indicates the threshold for genome-wide significance (posterior probability ≥0.5). Model 1 results are shown for A) SBP and B) DBP. Model 2/UT-Self plot for C) SBP and D) DBP. Model 2/UT-Other plot for E) SBP and F) DBP. Model 3/UT-Self No/Yes plot for G) SBP and H) DBP. Model 3/UT-Other No/Yes plot for I) SBP and J) DBP.

Model 1 joint admixture and association mapping detected eight significant SNPs for SBP (Fig 2A, Table 2 & S2 Table). Among the significant SNPs identified, SNP rs56766116 is located in the *LRP8* gene, SNPs rs6739240 and rs72783028 are located in the *CAPN13* gene, SNPs rs6791604 and rs2320172 are located in the *MITF* gene, SNP rs2116737 is located in the *SGCD* gene, and SNPs rs80149157 and rs67579183 are located in the *MLL3* gene. We detected three significant associations when testing DBP and all three SNPs (rs77804878, rs114805596, rs17173425) are located in the *MLL3* gene (Fig 2B, Table 3 & S3 Table).

**Table 2:** Significant SNPs associated with SBP from joint ancestry and association analyses.

Model	UT	SNP	Chr	Closest Gene	Joint posterior
Model 1	N/A	rs56766116	1	*LRP8*	1.00
		rs6739240	2	*CAPN13*	0.56
		rs72783028	2	*CAPN13*	1.00
		rs6791604	3	*MITF*	0.82
		rs2320172	3	*MITF*	0.88
		rs2116737	5	*SGCD*	0.52
		rs80149157	7	*MLL3*	1.00
		rs67579183	7	*MLL3*	0.98
Model 2	UT-Self	rs2116737	5	*SGCD*	0.64
	UT-Other	rs115805528	3	*MITF*	1.00
		rs7962445	12	*PRH1-PRR4*	0.98
Model 3 -	SNP*UT-Self	rs11190458	10	*PKD2L1*	0.58
UT variable coded as No/Yes	SNP*UT-Other	rs35283004	5	*HTR4/ADRB2*	0.56
		rs11042725	11	*SBF2/ ADM*	0.99
		rs547330	3	*ABI3BP*	0.62
Model 3 -	SNP*UT-	rs12050767	15	*CYP19A1*	1.00
UT-Other coded as Low/High	Other	rs34712049	15	*CYP19A1*	1.00

Note: joint posterior probability ≥0.5 is considered significant

**Table 3:** Significant SNPs associated with DBP from joint ancestry and association analyses.

Model	UT	SNP	Chr	Closest Gene	Joint posterior
Model 1	N/A	rs77804878	7	MLL3	0.99
		rs114805596	7	MLL3	0.99
		rs17173425	7	MLL3	0.99
Model 2	UT-Self	rs2597955	12	PRH1-PRR4	0.99
		rs2600370	12	PRH1-PRR4	1.00
		rs2600368	12	PRH1-PRR4	0.99
		rs2708349	12	PRH1-PRR4	0.99
		rs2600362	12	PRH1-PRR4	0.99
		rs2597921	12	PRH1-PRR4	0.99
		rs2416545	12	PRH1-PRR4	0.99
		rs2445762	15	CYP19A1	0.874
	UT-Other	rs1319603	1	KCNK2/KCTD3	0.98
Model 3 -	SNP*UT-	rs11190458	10	PKD2L1	0.53
UT variable coded as No/Yes	Self				
Model 3 -	SNP*UT-	rs34712049	15	CYP19A1	1.00
UT-Other coded as Low/High	Other				

Note: joint posterior probability ≥0.5 is considered significant

Model 2 builds on Model 1 by including one of two measures of unfair treatment [unfair treatment of self (UT-Self) and unfair treatment of others (UT-Other)]. When testing SBP, we detected one significant association when including UT-Self (rs2116737 is located in the *SCGD* gene) (Fig 2C, Table 2 & S2 Table) and two significant associations when including UT-Other (rs115805528 in the *MITF* gene and rs7962445 in the *PRH1-PRR4* gene) (Fig 2E, Table 2 & S2 Table). When testing DBP, we detected eight significant associations when including UT-Self (rs2597955, rs2600370, rs2600368, rs2708349, rs2600362, rs2597921, rs2416545 located in *PRH1-PRR4* and rs2445762 located in *CYP19A1* gene) (Fig 2D, Table 3 & S3 Table) and one significant associations when including UT-Other (rs1319603 downstream of *KCNK2/KCTD3* genes) (Fig 2F, Table 3 & S3 Table).

**Fig 3:**
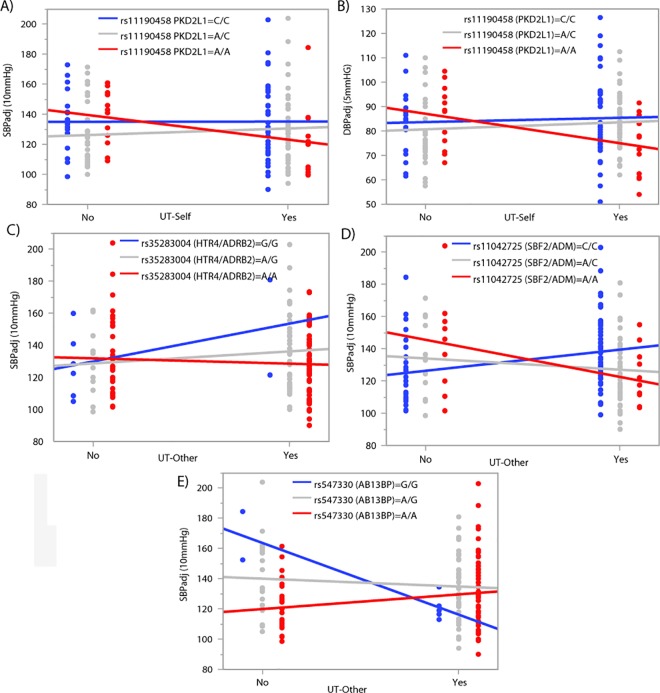
SNP x unfair treatment interaction effects associated with BP. BP levels are shown on the y-axis and unfair treatment (No/Yes) on the x-axis. SNP genotype is colored blue, gray or red. A) Significant association between SBP and UT-Self is dependent on SNP rs11190458 genotype in the *PKD2L1* gene. B) Significant association between DBP and UT-Self is dependent on SNP rs11190458 genotype in the *PKD2L1* gene. Significant associations between SBP and UT-Other are dependent on SNP genotypes C) rs35283004 upstream of *HTR4/ADRB2* genes D) rs11042725 upstream of *SBF2/ADM* genes and E) rs547330 in the *ABI3BP* gene.

Model 3 builds on the previous models by testing for interactions between SNP genotype and unfair treatment associated with BP. Unfair treatment measures were first dichotomized as No/Yes variables. For SBP, we identified one significant interaction with UT-Self (rs11190458 in *PKD2L1*) (Fig 2G, Table 2 & S2 Table) and three significant interactions with UT-Other (rs35283004 upstream of *HTR4* and *ADRB2*, rs11042725 upstream of *SBF2* and *ADM*, and rs547330 in *ABI3BP*) (Fig 2I, Table 2 & S2 Table). For DBP, we detected one significant association with UT-Self (rs11190458 in *PKD2L1*) (Fig 2H, Table 3 & S3 Table). The interactions between SNP genotype and level of unfair treatment associate with a range of BP readings including increased variation in BP at both low and high levels of unfair treatment depending on the gene (Fig 3).

We also dichotomized unfair treatment measures as Low/High and identified two significant interactions with UT-Other (rs12050767 and rs34712049 in *CYP19A1*) for SBP (S2A Fig, Table 2 & S2 Table) and one (rs34712049 in *CYP19A1*) for DBP (S2D Fig, Table 3 & S3 Table). Interactions with *CYP19A1* appear to be sex dependent, where men show more variation in BP at high levels of UT-Other dependent on *CYP19A1* genotype when compared to women (S2B, S2C and S2E Fig).

Most of the SNPs we identified to be significantly associated with BP are associated with genes that have been previously related to cardiovascular phenotypes (with the exception of PRH1-PRR4 that has no known associations) (Table 4). However, when we test for SNP by UT-Other interactions, we identify a new class of genes that have been related to psychological phenotypes, including psychosocial distress and mood disorders (Table 4, in italics).

**Table 4:** Comparison of the significant genes that we found to be associated with BP with other reported phenotypes.

Model	BP	Effect	Genes	Associated phenotype	Ref.
Model 1	SBP	SNP	LRP8	Triglyceride levels in early onset coronary artery disease	[40]
			CAPN13	BMI in African American girls	[41]
			MITF	Pulmonary hypertension	[42]
			SGCD	Hypertrophic cardiomyopathy	[43]
				HDL-cholesterol	[44]
				Coronary spastic angina	[45]
			MLL3	Regulation of Renin through HOXB9	[46]
	DBP	SNP	MLL3	Regulation of Renin through HOXB9	[46]
Model 2	SBP	UT-Self	SGCD	Hypertrophic cardiomyopathy	[43]
		UT-Other	MITF	Pulmonary hypertension	[42]
			PRH1-PRR4	No known associations	
	DBP	UT-Self	CYP19A1	SNP x Sex interactions and cardiovascular disease	[47]
			PRH1-PRR4	No known associations	
		UT-Other	*KCNK2/KCTD3*	Depressive disorders	[48–50]
Model 3	SBP	SNP*UT-Self	PKD2L1	Serum metabolites in African Americans	[51]
UT variable		SNP*UT- Other	*ABI3BP*	Suicide attempts among patients with depression	[52, 53]
coded as No/Yes			*HTR4*	Suicide attempts in schizophrenia patients	[51]
			*ADRB2*	Psychological distress	[54]
			*SBF2*	Addiction	[55]
			*ADM*	Anxiety, Depression, and Bi-polar disease	[56]
	DBP	SNP*UT-Self	PKD2L1	Serum metabolites in African Americans	[51]
		SNP*UT-Other	N/A		
Model 3	SBP	SNP*UT-Other	CYP19A1	SNP x Sex interactions and cardiovascular disease	[47]
UT variable coded as Low/High	DBP	SNP*UT-Other	CYP19A1	SNP x Sex interactions and cardiovascular disease	[47]

Note: italicized gene names have previously been related to psychological phenotypes

## Discussion

The etiology of many complex diseases, such as hypertension, is not well understood. Although genetic and environmental factors are important determinants of complex disease, they are rarely both included in genetic association studies. Specifically, the factors that influence BP, and the role these factors play in racial disparities in hypertension, are under debate. It is known that genetic susceptibility and exposure to sociocultural risk factors are both associated with hypertension and other complex diseases. Moreover, sociocultural stressors linked to systemic racism in the US may contribute to the racial disparities [57] seen with hypertension and other complex diseases in African Americans. Even with this knowledge, the majority of genetic studies of complex diseases performed to date include minimal sociocultural data.

Our study indicates that both genetic and sociocultural factors contribute to a comprehensive model of BP variation. Association and admixture analyses by themselves did not detect any significant SNPs. In contrast, our results from a joint admixture and association study of BP, tested in three models that sequentially incorporated sociocultural data, did identify significant associations. In particular, interactions between SNPs and UT-Other revealed a class of genes implicated in multiple phenotypes including psychosocial distress or mood disorders.

The eight significant SNPs identified in Model 1 are located in five genes (Fig 2)—*LRP8*, *CAPN13*, *MITF*, *SGCD* and *MLL3*—previously implicated in BP variation or cardiovascular disease. *LRP8* gene has been associated with triglyceride levels, coronary artery disease, and myocardial infarction [40]. *CAPN13* gene was significantly associated with a gene x BMI interaction effect for BP among African Americans [41]. *MITF* stimulates the transcription of *HIF1A* [58], which contributes to pulmonary hypertension [42]. *SGCD* is a risk factor for hypertrophic cardiomyopathy [43], HDL-cholesterol [44], and coronary spastic angina [45]. The histone methylase *MLL3* (associated with SPB and DBP) has been demonstrated to mediate *HOXB9* expression with estrogens [46], and *HOXB9* regulates renin, an enzyme that influences BP [59].

In Model 2, we expanded Model 1 to include sociocultural data, specifically measures of unfair treatment. Model 2 replicated associations at two genes from Model 1 (*SGCD* and *MITF*) and identified a novel locus of unknown function (*PRH1-PRR4*). A SNP in the *CYP19A1* gene was identified to be associated with DBP when UT-Self was included in Model 2. The *CYP19A1* gene converts androgens to estrogens and a significant interaction between *CYP19A1* genotype and sex on cardiovascular outcomes was previously identified [47]. Lastly, a SNP downstream of the *KCNK2* was associated to DBP in Model 2 that included UT-Other. The *KCNK2* gene encodes a K+ channel and has been associated with adrenal aldosterone-producing adenomas [50], hereditary hypertension [50], and depressive disorders [48, 49].

In Model 3, we tested for interactions between SNPs and unfair treatment that were associated with BP. The “weathering hypothesis” proposes a threshold of cumulative psychosocial distress over the life course before effects on physical and biological health are observed [60]. We dichotomized the unfair treatment variables in two ways (No/Yes; Low/High) in order to model different thresholds at which the weathering hypothesis might manifest in our study. We identified one interaction effect between a SNP in the *PKD2L1* gene and UT-Self in association with SBP and DBP. *PKD2L1* is a non-selective calcium ion regulation channel and has been associated with metabolites in serum among African Americans [51].

When we tested for interactions between UT-Other and SNPs in association with BP, we identified six interaction effects in genes that are associated with multiple psychological phenotypes, including psychosocial distress and mood disorders (Table 4). These effects included an interaction between UT-Other and a SNP upstream of genes *HTR4/ADRB2; HTR4* was previously associated with schizophrenia and suicide attempts [53], and *ADRB2* was associated with psychological distress [54]. Another interaction was between UT-Other and a SNP upstream of the *SBF2/ADM* genes; *SBF2* was associated with addiction [55], and *ADM* was associated with anxiety [61], depression and bipolar disease [56]. We also found that UT-Other interacts with a SNP in gene *ABI3BP* that was previously associated with suicide attempts among patients with depression [52]. The interaction effects between SNPs and UT on BP are varied and difficult to explain in detail. However, there is evidence that some SNPs may contribute to an increase in BP at high levels of UT, e.g. HTR4/ADRB2 A/A homozygotes, while others appear to be protective at high levels of UT, e.g. PKD2L1 A/A homozygotes. Although not significant on its own, UT exposure may be necessary to reveal the SNP effect (protective or otherwise) as is seen in some studies of mental illness in which exposure to early life adversity is necessary to reveal its interaction with the risk variant, e.g. serotonin transporter SNP, *5-HTTLPR* [62].

Finally, we identified interactions between UT-Other and three SNPs in the *CYP19A1* gene located on chromosome 15 to be associated with SBP and DBP. *CYP19A1* encodes aromatase, the enzyme responsible for the conversion of androgens to estrogens, and may play a role in variation in outcomes among men and women with cardiovascular disease. A significant interaction had been previously identified between *CYP19A1* SNP genotypes and sex that increased cardiovascular related mortality in men and decreased cardiovascular related mortality in women [47]. Based on these results, we tested for a sex effect in the UT-Other*CYP19A1 SNPs and observed suggestive evidence that the interactions between UT-Other and genotypes of *CYP19A1* SNPs associated with BP are dependent on sex, i.e. BP varies more at high levels of unfair treatment of others based on *CYP19A1* SNP genotypes in men when compared to women (S3 Fig). If confirmed in future studies, these results suggest that some *CYP19A1* variants may have a protective effect and some may increase vulnerability to hypertension based on exposure to UT-Other and sex. Furthermore, these results indicate that both genetic and sociocultural factors contribute to a comprehensive model of BP variation.

The cumulative effect of multiple genetic risk factors and an individual’s susceptibility to sociocultural stressors is complex. It makes sense that genes involved in mood disorders, such as depression, suicide and addiction, might be involved in the interactions between stress from discrimination, genetic risk factors, and BP in African Americans, and that the interactions might be circular and feedback on each other. For example, chronic illnesses can lead to depression [63], and recent studies also indicate that depression in otherwise healthy people may increase their risk of developing heart disease [64]. Furthermore, chronic exposure to stress (such as discrimination) or the stress of poor health may be a risk factor for depression. Finally, a single genetic risk factor may be associated with multiple complex diseases. Since we used a cross-sectional design, it is not possible to disentangle the causal relationship between stress, disease, genetic risk factors and BP in our study. However, we can speculate on scenarios in which these factors may interact to influence health. Everyday encounters with discrimination are causally associated with poor mental health outcomes in African Americans [65], which could then lead to the development of increased BP. The extent to which the experience of systemic racism is unique to African Americans may help explain the racial disparities seen in hypertension, and possibly other complex diseases.

We find evidence that UT-Other is a salient risk factor for BP in our study population. Furthermore, UT-Other reveals novel biological processes involved in hypertension and possibly involved in racial health disparities. There are several reasons why experiences with UT-Other and UT-Self may differ in their effect on blood pressure. As we previously described [29], UT-Self neglects social and cultural context and may be biased by participants’ reluctance to report personal experiences of unfair treatment. Denying that one has experienced discrimination could itself be a coping strategy to avoid stigma, and the physiological consequences of this strategy are unclear. Furthermore, people may feel greater distress from hearing about bad experiences of people close to them over which they have little control. It is also possible that hearing about friends’, family members’, or coworkers’ experiences of unfair treatment may prime people to anticipate the same experience in similar social settings. The resulting state of hypervigilance may be uniquely associated with elevated risk of hypertension in African Americans [66]. Regardless, the key consideration is that a physiological stress response can arise without direct experience of the stressor, i.e. the stress response can be activated by observing an individual experience a stressful situation [67]. We are most likely under-estimating the stress associated with unfair treatment since we do not capture less proximate experiences such as those reported in the news and social media, including shootings of unarmed black men and women, which clearly contribute to African American distress.

Multiple studies have discussed the “missing heritability” of complex phenotypes [30–32]. While most studies focus on the need for more samples, more genetic markers, better genetic models or analytic methods, the consistent factor is that heritability is “missing” only for complex diseases. By definition, complex diseases are caused by multiple genetic and environmental factors. Our current and previous results [20, 29, 68] suggest that interactive effects between genetic and environmental factors may account for part of the missing heritability. Both types of data must be included in order to detect interactive effects. Omitting sociocultural data when the biological effects of genetic variants act through an interaction with the sociocultural environment makes it impossible to properly interpret the genetic effects. Thus, the lack of replication in genetic studies of hypertension could be due to a lack of sociocultural stressor data and inability to test for gene by environment (G x E) interactions.

Our study highlights how a biocultural approach can provide a more complete understanding of a complex disease, such as hypertension. Using a joint analysis that includes ancestry and genetic data, we replicate previously identified loci for BP variation. More importantly, we show that inclusion of both genetic and sociocultural data allows the identification of significant interaction effects. In our study, a novel measure of discrimination, UT-Other = unfair treatment of others, suggests that a new class of genes associated with psychosocial distress and mood disorders may be particularly relevant to the manifestation of hypertension in African Americans and underlying racial disparities. The unfair treatment measures we capture in our study are specific to African Americans, but our results may be relevant to other groups who experience similar discrimination from unfair treatment. The differences we identified between UT-Other and UT-Self suggest that the biological consequences of discrimination experiences are complex and not well understood. Further study is warranted to better understand the effects that genes and social stressors, like discrimination, have on disease outcomes and that may contribute to persistent racial disparities in health.

## Methods

### Ethics Statement

As part of a community based participatory research approach, a local steering committee (Health Equity Alliance of Tallahassee Steering Committee, Tallahassee, Florida) was formed to advise on all aspects of the project including informed consent and data dissemination. The research protocol was also approved by the University of Florida Institutional Review Board (IRB-01 (#364–2008) and IRB-02 (#2007-U-469)). Informed written consent was obtained from all participants prior to data collection. Due to the sensitive nature of the sociocultural data and the potentially identifiable genetics data, the informed consent requires that the genetic and sociocultural data be made available through our steering committee. The members of the steering committee and their contact information are as follows:primary contact—Qasimah Boston: abarakaq@aol.com, James Bellamy: bellemyjhsd@embarqmail.com, Edward Holifield: ewholifield@yahoo.com, Miaisha Mitchell: mmiaisha@gmail.com, Cynthia Seaborn: cynthia.seaborn@gmail.com.

### Research setting and sample population

Data were collected in Tallahassee, Florida from adult African Americans who were selected using a multistage probability sampling design. Stratification was first performed by Census block groups using cluster analysis of neighborhood-level indicators of racial composition and material deprivation to maximize contrasts in relevant social stressors. Next, within each cluster, block groups were randomly selected and then residential addresses within sampled block groups were chosen. Last, one participant from among eligible adults (self-identified African American, age 25–65) in each household was selected. The final number of individuals was 157 with complete data, which included buccal swabs for DNA analysis and collection of sociocultural and biological data during a face-to-face interview.

### Saliva Samples

Saliva samples were collected and stored using Oragene DNA Collection Kits (DNA Genotek, Ontario, Canada). DNA was extracted according to the manufacturer’s protocol.

### Genotyping data

More than 30,000 SNPs and ancestry informative markers (AIMs) on all 22 autosomal chromosomes were genotyped using a custom Affymetrix Axiom Array that we designed (Affymetrix, Santa Clara, CA). These SNPs were chosen because they were located within or near genes that previously had been found to associate with hypertension, cardiac disease, stress, and pigmentation phenotypes in other studies and on existing Affymetrix arrays (see S4 Table for a list of the 30,000+ SNPs and associated genes and other attributes). AIMs were identified from the Illumina African American Admixture panel (Illumina, San Diego, CA). We followed the recommendations set by Affymetrix’s Axiom Genotyping Console to call all SNP genotypes that had a genotyping success call rate ≥ 97%. SNP marker properties (minor allele frequency (MAF), call rate, Hardy-Weinberg Equilibrium (HWE) test) were calculated using PLINK [69] and JMP/Genomics (SAS 9.4). Only SNPs with a MAF >1%, a call rate >95%, and SNPs that were in HWE were included (p-value >0.001 for HWE test). This resulted in a final set of 27,559 SNPs and 3,197 AIMs for further analysis.

### Sociocultural and biological data

Sociocultural data were obtained using a survey that was developed based on the initial ethnographic phase of the project. In the ethnographic interviews, many respondents narrated their experiences of discrimination by citing unfair treatment experienced by someone close to them, e.g. close friends, family, and coworkers. Using this information, we adapted a standard measure of perceived discrimination [70, 71] that usually asks only about respondents' own experiences in nine social domains (e.g., police, courts, housing), to include if "you or someone close to you" had been treated unfairly in each domain. If participants answered "yes", we subsequently asked who had experienced the unfair treatment given the following options—you, your spouse, your parents, your child, your grandchild, a sibling, another relative, or a close friend. Unfair treatment was measured as an unweighted count of affirmative answers to the questions of unfair treatment in nine social domains with a maximum theoretical value of nine. The discrimination scales included: any/major instances of unfair treatment experienced by the study participant during their lifetime or experienced by individuals close to the study participant (range of 0–9).

Biological data were also obtained. Weight and height were measured using a digital scale and portable stadiometer and used to calculate body mass index (BMI) (weight in kg/height in m^2^). Data on age, sex, years of education and antihypertensive medication use were collected. Three resting blood pressure readings were taken at the beginning of the interview using a standardized protocol in which respondents were seated for approximately 30 minutes and had not consumed alcohol or tobacco. Blood pressure was measured using an oscillometric monitor (UA-787, A&D Medical, Tokyo, Japan) that had been validated according to the European Society of Hypertension protocol [72]. The first readings differed significantly from the latter two readings and thus an average of the latter two readings was used in the study analyses. Adjusting for BP medications taken has been shown to be more powerful than fitting a conventional regression model with treatment as a binary covariate [73]. Thus, we corrected for antihypertensive medication use by adding 10mmHg to the mean SBP [73] and 5 mmHg to the mean DBP readings [74] of each participant who was taking antihypertensive medication. The mean adjusted SBP and DBP measurements were treated as dependent variables in our analysis.

### Inferring Local and Global Ancestry

Each SNP genotype was coded as 0, 1, or 2 copies of the derived allele and used to infer local ancestry at each SNP (n = 30,756) by running LAMP [75], a method for estimating ancestries at each locus in a population of admixed individuals. All 30,756 SNPs, including 3,197 AIMs, were used to infer local ancestry. We used the default parameters set in LAMP, with the number of populations set to 2, the number of generations set to 7, and the mixture proportions alpha set to 0.8 African, 0.2 European. The output local ancestry was coded as 0, 1, or 2 copies of inherited African ancestry. The mean global African Ancestry was estimated by averaging local ancestry across loci for each individual.

### Estimating kinship and relatedness

We used PC-AiR [76], *Principal Component Analysis in Related* samples, to account for relatedness in our sample. PC-AiR analysis was run in R using kinship estimates obtained from the software KING [77] for the 157 participants and 26,343 SNP genotypes that had a minor allele frequency (MAF) ≥ 5%. PC-AiR identified 120 unrelated individuals and 37 related individuals in our sample with a kinship coefficient threshold of 0.025. The top ten PC-AiRs were included in the admixture and association mapping as covariates to account for relatedness.

### Admixture Mapping

Since the underlying genetic susceptibility of hypertension may be different for elevated SBP and DBP, we chose to analyze each set of BP measurements separately.

We performed admixture mapping by linearly regressing blood pressure on local ancestry, adjusted for global ancestry, sex, age, education, BMI and relatedness (PCAiRs_1-10_) in the following model:

Admixture mapping:

$${SBP}_{adj}=\mu +local\;ancestry+global\;ancestry+sex+age+education+BMI+PC{AiR}_{1–10}+\varepsilon$$

$${DBP}_{adj}=\mu +local\;ancestry+global\;ancestry+sex+age+education+BMI+PC{AiR}_{1–10}+\varepsilon$$

where ε is assumed to be normally distributed with a mean of zero.

### Association Mapping

We performed an association study for blood pressure by linearly regressing blood pressure on SNP genotype, adjusted for global ancestry, sex, age, education, BMI and relatedness (PCAiRs_1-10_) in the following model:

Association mapping:

$${SBP}_{adj}=\mu +SNP+global\;ancestry+sex+age+education+BMI+PC{AiR}_{1–10}+\varepsilon$$

$${DBP}_{adj}=\mu +SNP+global\;ancestry+sex+age+education+BMI+PC{AiR}_{1–10}+\varepsilon$$

where ε is assumed to be normally distributed with a mean of zero.

### Joint Admixture and Association Mapping

We used Bayesian joint admixture and association mapping with partial Bonferroni correction [36] to identify SNPs that were associated with BP using posterior probabilities from admixture mapping as the prior probabilities for association mapping. We performed joint admixture and association mapping for mean adjusted SBP and DBP by stratifying based on local ancestry, linearly regressing blood pressure on local ancestry, adjusting for global ancestry, sex, age, education, BMI and genetic relatedness (PCAiRs_1-10_), and recombining the p-values while adjusting for inverse variance-weighted fixed effects. Due to lack of variance for 1446 SNP genotypes when stratified by ancestry, we excluded these SNPs from the joint admixture and association mapping.

We developed three progressive models that assessed the association between SNP and BP (Model 1), SNP and BP including measures of unfair treatment (Model 2), and SNP and BP including interaction effects between SNP x UT (Model 3).

Model 1:

$${SBP}_{adj}=\mu +SNP+global\;ancestry+sex+age+education+BMI+PC{AiR}_{1–10}+\varepsilon$$

$${DBP}_{adj}=\mu +SNP+global\;ancestry+sex+age+education+BMI+PC{AiR}_{1–10}+\varepsilon$$

where ε is assumed to be normally distributed with a mean of zero.

In Model 2, we performed the same joint admixture and association mapping as in Model 1 with unfair treatment measures added to the model.

Model 2:

$${SBP}_{adj}=\mu +SNP+global\;ancestry+sex+age+education+BMI+UT+PC{AiR}_{1–10}+\varepsilon$$

$${DBP}_{adj}=\mu +SNP+global\;ancestry+sex+age+education+BMI+UT+PC{AiR}_{1–10}+\varepsilon$$

where ε is assumed to be normally distributed with a mean of zero.

In Model 3, we included an interaction term between SNP genotype and unfair treatment by performing the same joint admixture and association mapping as in Model 2 with the interaction term added to the model.

Model 3:

$${SBP}_{adj}=\mu +SNP+global\;ancestry+sex+age+education+BMI+UT+SNP\times UT+PC{AiR}_{1–10}+\varepsilon$$

$${DBP}_{adj}=\mu +SNP+global\;ancestry+sex+age+education+BMI+UT+SNP\times UT+PC{AiR}_{1–10}+\varepsilon$$

where ε is assumed to be normally distributed with a mean of zero.

We were also interested in testing the “weathering hypothesis,” which proposes a threshold of experienced discrimination before effects on physical and biological health are observed [60], so we dichotomized the unfair treatment measures to test in Model 3. We dichotomized in two ways: No (0 affirmative responses to experiences of unfair treatment questionnaire) versus Yes (≥1 affirmative responses), as has been done in other studies [26]; and Low (0–2 affirmative responses to experiences of unfair treatment questionnaire) versus High (≥ 3) experience of unfair treatment. We based our cut-off for low versus high experience of unfair treatment on our previous work where a threshold effect of unfair treatment on blood pressure was seen at UT-Other ≥ 3 [29]. In order to reduce the effect of outlier values, we further filtered SNP genotypes and included only SNPs with a MAF > 10%. This MAF threshold was chosen in order to have a reasonable sample size of individuals homozygous for the minor allele for each level of UT in order to test for interaction effects. This resulted in a final set of 18,324 SNPs. The R code is provided for the admixture mapping, association mapping and the joint analyses.

### Setting significance thresholds

Admixture and association studies were first performed using a frequentist approach. Bonferroni correction was applied in order to correct for multiple testing by dividing the significance threshold alpha (0.05) by each analysis’ testing burden (the number of tests performed). For admixture mapping, we estimated the effective number of tests for each chromosome for each individual by summing the number of local ancestry switches that occur. We summed the effective number of tests for the 22 autosomal chromosomes for each individual and then averaged across individuals. We used this average number of ancestry switches to correct for multiple testing in admixture mapping. Thus, we set our significance threshold for admixture analysis to be -log10 (p-value) = -log10 (0.05/78.82) = 3.2. For association mapping, we applied Bonferroni correction to account for the number of SNPs tested in our analysis that passed the quality control thresholds described previously. Thus, we set our significance threshold for association mapping to be–log 10 (p-value) = -log10 (0.05/27,559) = 5.74.

The joint admixture and association analysis used a Bayesian approach to test for association between genotype and BP using local ancestry as our prior. The prior probability is dependent on the degree of African ancestry, with more weight given to an association with a higher level of African ancestry. In the joint analysis, the testing burdens calculated for admixture and association analysis were accounted using partial Bonferroni testing. The significance of association for the joint analysis was reported by the joint posterior probability (0–1), with a value of 1 indicating the highest probability that a SNP is associated to the phenotype. The genome-wide significance level for the joint posterior probability was set to 0.5, with values equal to or greater than 0.5 being considered as associated with BP.

### Simulation of type I error rate

Any significant association identified in any joint model that had a joint posterior ≥ 0.5 was simulated 10,000 times to obtain the false positive rate by randomly re-shuffling the associated blood pressure readings and covariates, which were kept linked to one another, to the SNP genotype and ancestry estimates, using the joint admixture and association code. We kept genotype and ancestry data fixed and shuffled the phenotype and covariate data for each participant. We set our seed to 385 in R for the simulations. We plotted the distributions of the simulations and determined that any SNP that reached a posterior prior of ≥0.5, 500 times or fewer in the 10,000 simulations was considered a true positive hit (since 500/10,000 = 0.05). In our results, we only report the significant SNPs that have been confirmed through simulations for the joint admixture and association analyses.

## Supporting Information

S1 Table Association between BP and covariates. (PDF)

S2 Table Significant SNPs associated with SBP identified from the joint ancestry and association analysis (Expanded version of Table 2). (PDF)

S3 Table Significant SNPs associated with DBP identified from the joint ancestry and association analysis. (Expanded version of Table 3).
(PDF)

S4 Table List of SNPs on custom Affymetrix Axiom Array that we used in current analysis. These SNPs were chosen because they were located within or near genes that previously had been found to associate with hypertension, cardiac disease, stress, and pigmentation phenotypes in other studies and on existing Affymetrix arrays (30,756 SNPs and associated genes and other attributes).
(CSV)

S1 Fig Manhattan plot of frequentist admixture mapping for A) SBP and B) DBP. Each association is plotted based on its chromosomal position (x-axis) and its significance (-log10 (pval) plotted on the y-axis). The threshold for significance was set to -log10 (pval) = 3.20 (dashed line). Manhattan plot of frequentist association mapping for C) SBP and D) DBP. Each association is plotted based on its chromosomal position (x-axis) and its significance (-log10 (pval) plotted on the y-axis). The threshold for significance was set to -log10 (pval) = 5.74 (dashed line).
(TIF)

S2 Fig SNP x UT-Other Low/High interactions. A) Bayesian Manhattan plot for joint ancestry and association testing with SBP for Model 3 that tests for interactions between SNP and UT-Other Low/High. The y-axis indicates the posterior probability that a locus affects BP. The dashed line indicates the threshold for genome-wide significance (posterior probability = 0.5). Two SNPs in the CYP19A1 gene reached significance. B) Plot of the SNP rs12050767 genotype x UT-Other interaction effect associated with SBP. SBP levels are shown on the y-axis and unfair treatment (Low/High) on the x-axis. SNP genotype is colored red, gray and blue. C) Plot of the SNP rs34712049 genotype x UT-Other interaction effect associated with SBP. SBP levels are shown on the y-axis and unfair treatment (Low/High) on the x-axis. SNP genotype is colored red, gray and blue. D) Bayesian Manhattan plot for joint ancestry and association testing with DBP for Model 3 that tests for interactions between SNP and UT-Other Low/High identified 1 SNP in the CYP19A1 gene. E) Plot of the SNP rs34712049 genotype x UT-Other interaction effect associated with DBP. DBP levels are shown on the y-axis and unfair treatment (Low/High) on the x-axis. SNP genotype is colored red, gray and blue.
(TIF)

S3 Fig SNP x UT-Other interaction effects associated with BP appear to be sex dependent. A-C) Association between SNPs in the CYP19A1 gene are associated with BP in a sex dependent manner. BP levels are shown on the y-axis and SNP genotypes are plotted on the x-axis. Individuals are colored by sex. D-I) Association between UT-Other Low/High and BP are dependent on CYP191A genotypes and are plotted separately for men and women. BP levels are shown on the y-axis and unfair treatment (Low/High) on the x-axis. SNP genotype is colored green, purple or red. Graphs are plotted separately for men and women. D-E) Significant association between SBP and UT-Other is dependent on SNP rs12050767 genotype in the *CYP19A1* gene and sex. F-G)) Significant association between SBP and UT-Other is dependent on SNP rs34712049 genotype in the *CYP19A1* gene and sex. H-I) Significant association between DBP and UT-Other is dependent on SNP rs34812049 genotype in the *CYP19A1* gene and sex.
(TIF)

S1 File R code for the admixture mapping, association mapping, joint analyses and simulation analyses. (DOCX)
