# Supplementary material for: Genetic Loci and Novel Discrimination Measures Associated with Blood Pressure Variation in African Americans Living in Tallahassee
Source: PLoS One. 2016 Dec 21;11(12):e0167700. doi: 10.1371/journal.pone.0167700 (PMC5176163; doi:10.1371/journal.pone.0167700)
Supplement: S1 Table — (PDF) [file pone.0167700.s001.pdf]

Covariate	SBPadj	DBPadj
Global African Ancestry	0.5331	0.7272
Age	<0.0001	0.0009
Sex	0.0433	0.8792
BMI	<0.0001	<0.0001
Education (years)	0.2661	0.3292
UT-Self (0-9)	0.1499	0.3972
UT-Other (0-7)	0.5826	0.7857
UT-Self No/Yes	0.9844	0.9994
UT-Other No/Yes	0.7302	0.566
UT-Self Low/High	0.1683	0.9432
UT-Other Low/High	0.2146	0.4337
PC-AiR1	0.2658	0.7324
PC-AiR2	0.6153	0.9401
PC-AiR3	0.7383	0.294
PC-AiR4	0.2806	0.1268
PC-AiR5	0.9969	0.6097
PC-AiR6	0.8727	0.8387
PC-AiR7	0.2654	0.1801
PC-AiR8	0.0012	0.0957
PC-AiR9	0.2106	0.1192
PC-AiR10	0.2319	0.1726
