# Supplementary material for: Genetic Loci and Novel Discrimination Measures Associated with Blood Pressure Variation in African Americans Living in Tallahassee
Source: PLoS One. 2016 Dec 21;11(12):e0167700. doi: 10.1371/journal.pone.0167700 (PMC5176163; doi:10.1371/journal.pone.0167700)
Supplement: S2 Table — (PDF) [file pone.0167700.s002.pdf]

Model	UT	SNP	Chr	Position (bp)	Type	Assoc. Gene	MAF	Local AA**	Admix posterior	GWAS posterior	Joint posterior
Model 1	N/A	rs56766116	1	53757257	Intronic	*LRP8*	0.04	0.69	0.055	0.99	1.00
		rs6739240	2	30978800	Intronic	*CAPN13*	0.26	0.70	7.021E-5	0.39	0.56
		rs72783028	2	30980259	Intronic	*CAPN13*	0.01	0.70	7.021E-5	0.99	1.00
		rs6791604	3	69848416	Intronic	*MITF*	0.08	0.64	2.21E-6	0.99	0.82
		rs2320172	3	69916637	Intronic	*MITF*	0.14	0.64	2.21E-6	0.99	0.88
		rs2116737	5	155450424	Intronic	*SGCD*	0.30	0.87	0.022	0.0018	0.52
		rs80149157	7	151911409	Intronic	*MLL3*	0.07	0.67	4.36E-6	0.99	1.00
		rs67579183	7	152086858	Intronic	*MLL3*	0.08	0.66	4.64E-6	0.99	0.98
Model 2	UT-Self	rs2116737	5	155450424	Intronic	*SGCD*	0.30	0.87	0.021	0.0031	0.64
	UT-Other	rs115805528	3	69953514	Intronic	*MITF*	0.04	0.64	1.97E-6	0.99	1.00
		rs7962445	12	11305844	Intronic	*PRH1-PRR4*	0.36	0.36	0.0054	0.29	0.98
Model 3	SNP*UT-Self	rs11190458	10	102053992	Intronic	*PKD2L1*	0.39	0.70	0.0011	0.064	0.58
No/yes	SNP*UT-Other	rs35283004	5	1.5E+08	Upstream	*HTR4 ADRB2*	0.22	0.86	2.26E-6	0.97	0.56
		rs11042725	11	10325325	Upstream	*SBF2 ADM*	0.31	0.80	0.0027	0.80	0.99
		rs547330	3	100485785	Intronic	*ABI3BP*	0.24	0.85	0.000014	0.86	0.62
Model 3	SNP*UT-Other	rs12050767	15	51557257	Intronic	*CYP19A1*	0.38	0.69	0.000030	1.00	1.00
Low/High		rs34712049	15	51560064	Intronic	*CYP19A1*	0.27	0.69	0.000030	1.00	1.00

*Note: posterior probability ≥0.5 is significant

**local AA = mean local African Ancestry
