# Supplementary figures and images for: Genetic Loci and Novel Discrimination Measures Associated with Blood Pressure Variation in African Americans Living in Tallahassee
Source: PLoS One. 2016 Dec 21;11(12):e0167700. doi: 10.1371/journal.pone.0167700 (PMC5176163; doi:10.1371/journal.pone.0167700)

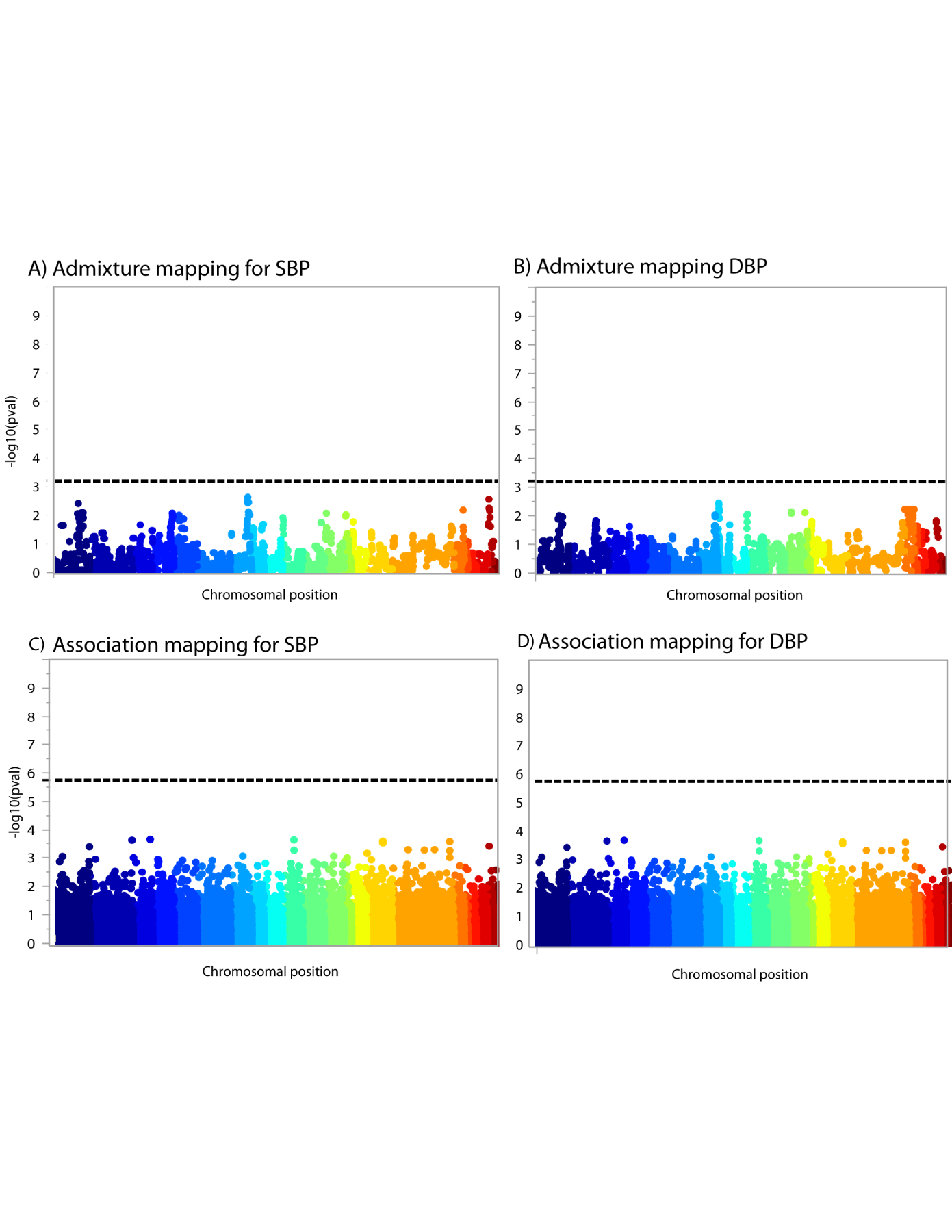

Supplement: S1 Fig — Manhattan plot of frequentist admixture mapping for A) SBP and B) DBP. Each association is plotted based on its chromosomal position (x-axis) and its significance (-log10 (pval) plotted on the y-axis). The threshold for significance was set to -log10 (pval) = 3.20 (dashed line). Manhattan plot of frequentist association mapping for C) SBP and D) DBP. Each association is plotted based on its chromosomal position (x-axis) and its significance (-log10 (pval) plotted on the y-axis). The threshold for significance was set to -log10 (pval) = 5.74 (dashed line). (TIF) [file pone.0167700.s005.TIF]

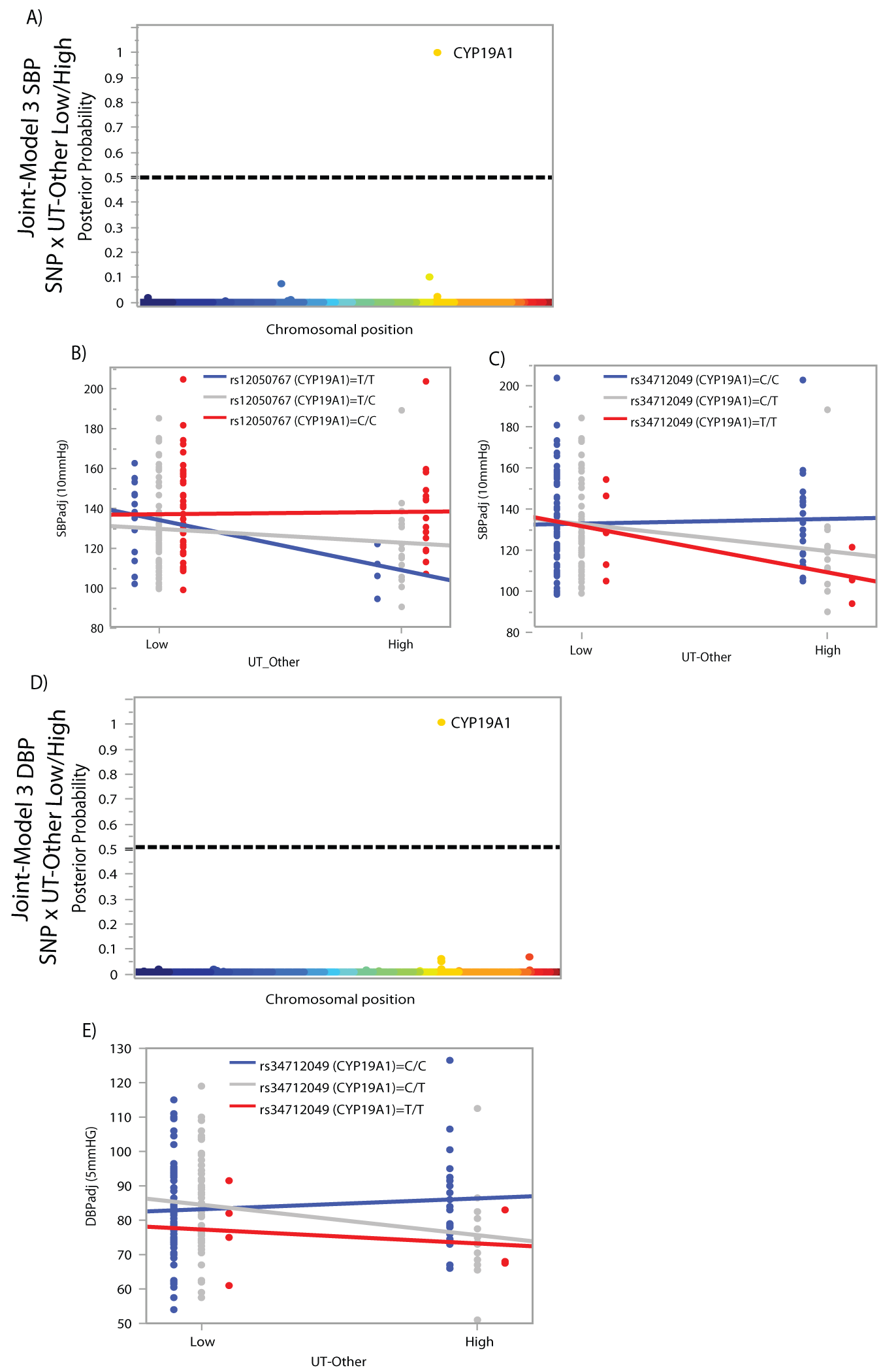

Supplement: S2 Fig — A) Bayesian Manhattan plot for joint ancestry and association testing with SBP for Model 3 that tests for interactions between SNP and UT-Other Low/High. The y-axis indicates the posterior probability that a locus affects BP. The dashed line indicates the threshold for genome-wide significance (posterior probability = 0.5). Two SNPs in the CYP19A1 gene reached significance. B) Plot of the SNP rs12050767 genotype x UT-Other interaction effect associated with SBP. SBP levels are shown on the y-axis and unfair treatment (Low/High) on the x-axis. SNP genotype is colored red, gray and blue. C) Plot of the SNP rs34712049 genotype x UT-Other interaction effect associated with SBP. SBP levels are shown on the y-axis and unfair treatment (Low/High) on the x-axis. SNP genotype is colored red, gray and blue. D) Bayesian Manhattan plot for joint ancestry and association testing with DBP for Model 3 that tests for interactions between SNP and UT-Other Low/High identified 1 SNP in the CYP19A1 gene. E) Plot of the SNP rs34712049 genotype x UT-Other interaction effect associated with DBP. DBP levels are shown on the y-axis and unfair treatment (Low/High) on the x-axis. SNP genotype is colored red, gray and blue. (TIF) [file pone.0167700.s006.tif]

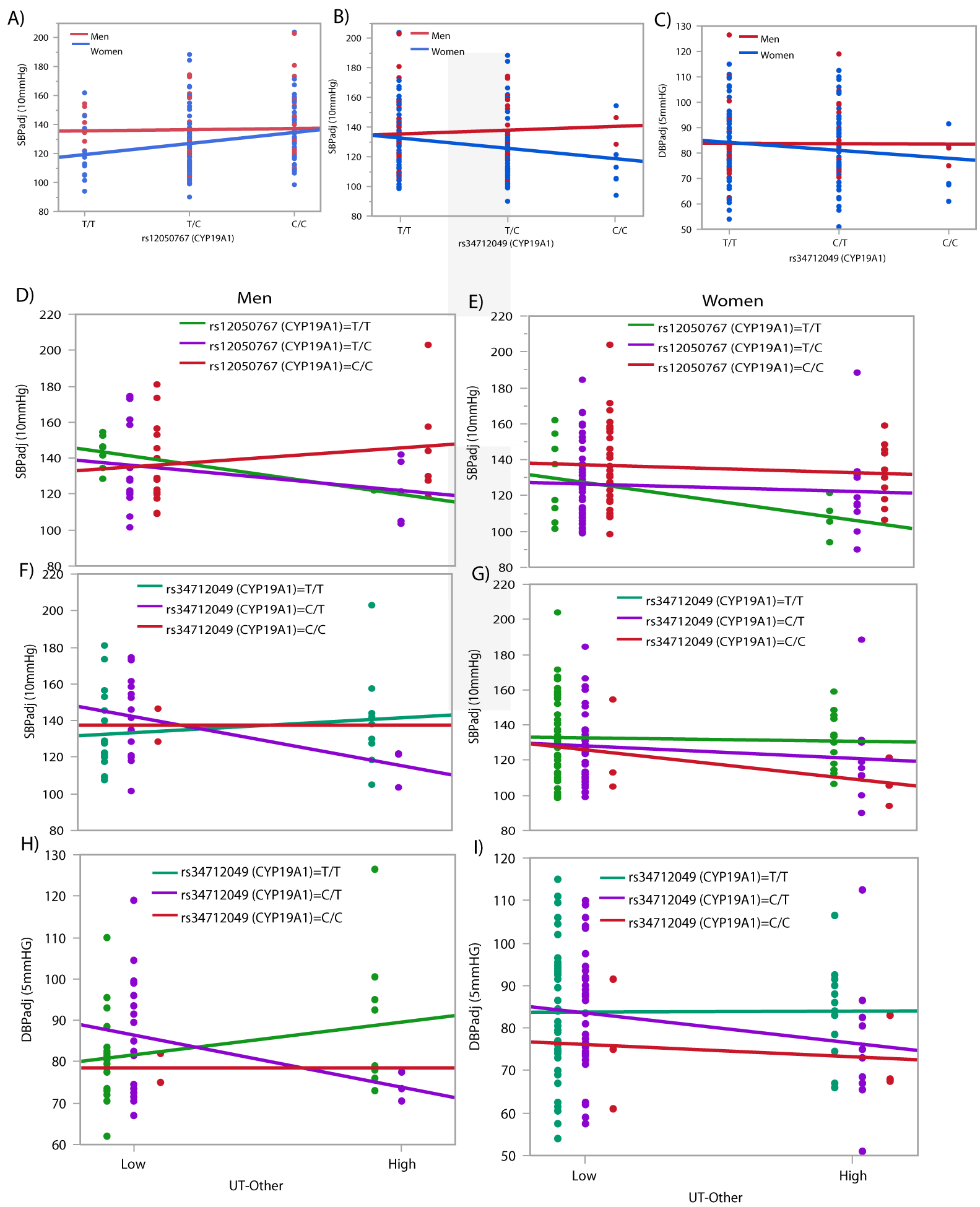

Supplement: S3 Fig — A-C) Association between SNPs in the CYP19A1 gene are associated with BP in a sex dependent manner. BP levels are shown on the y-axis and SNP genotypes are plotted on the x-axis. Individuals are colored by sex. D-I) Association between UT-Other Low/High and BP are dependent on CYP191A genotypes and are plotted separately for men and women. BP levels are shown on the y-axis and unfair treatment (Low/High) on the x-axis. SNP genotype is colored green, purple or red. Graphs are plotted separately for men and women. D-E) Significant association between SBP and UT-Other is dependent on SNP rs12050767 genotype in the CYP19A1 gene and sex. F-G)) Significant association between SBP and UT-Other is dependent on SNP rs34712049 genotype in the CYP19A1 gene and sex. H-I) Significant association between DBP and UT-Other is dependent on SNP rs34812049 genotype in the CYP19A1 gene and sex. (TIF) [file pone.0167700.s007.TIF]
