# Supplementary material for: Genetic Loci and Novel Discrimination Measures Associated with Blood Pressure Variation in African Americans Living in Tallahassee
Source: PLoS One. 2016 Dec 21;11(12):e0167700. doi: 10.1371/journal.pone.0167700 (PMC5176163; doi:10.1371/journal.pone.0167700)
Supplement: S1 File — (DOCX) [file pone.0167700.s008.docx]

#################

#################

#Admixture mapping

#from Jacklyn Quinlan et al

#Sept, 2015

#################

#################

localanc1<-

read.table(file="27559_LAMPancestry_estimates_MAF0.01,_HWE3_MISS0.05_code

0_1_2.tsv", header=F,sep="\t", na.strings="NA")

phenotype<-read.table(file="157_sbp10.tsv", header=F,sep="\t",

na.strings="NA")

GlobalMeanAncestry<-read.table(file="157_GA.tsv", header=F,sep="\t",

na.strings="NA")

sex<-read.table(file="157_sex.tsv", header=F,sep="\t", na.strings="NA")

age<-read.table(file="157_age.tsv", header=F,sep="\t", na.strings="NA")

bmi<-read.table(file="157_bmi.tsv", header=F,sep="\t", na.strings="NA")

edyears<-read.table(file="157_edyears.tsv", header=F,sep="\t",

na.strings="NA")

pc1<-read.table(file="157_pcair1.tsv", header=F,sep="\t",

na.strings="NA")

pc2<-read.table(file="157_pcair2.tsv", header=F,sep="\t",

na.strings="NA")

pc3<-read.table(file="157_pcair3.tsv", header=F,sep="\t",

na.strings="NA")

pc4<-read.table(file="157_pcair4.tsv", header=F,sep="\t",

na.strings="NA")

pc5<-read.table(file="157_pcair5.tsv", header=F,sep="\t",

na.strings="NA")

pc6<-read.table(file="157_pcair6.tsv", header=F,sep="\t",

na.strings="NA")

pc7<-read.table(file="157_pcair7.tsv", header=F,sep="\t",

na.strings="NA")

pc8<-read.table(file="157_pcair8.tsv", header=F,sep="\t",

na.strings="NA")

pc9<-read.table(file="157_pcair9.tsv", header=F,sep="\t",

na.strings="NA")

pc10<-read.table(file="157_pcair10.tsv", header=F,sep="\t",

na.strings="NA")

headers<-read.csv(file="header.csv",sep=",")

Output<-matrix(nrow=3,ncol=ncol(headers))

colnames(Output)<-colnames(headers)

rownames(Output) <- c("b", "se", "pval")

#phenotype is the vector of phenotypes

#localanc is the vector of local ancestry estimates at a marker

for (n in (1:27559)){

localanc<-c(localanc1[[n]])

y<-c(phenotype)

globalanc<-c(GlobalMeanAncestry)

result <-

summary(glm(y$V1~localanc+globalanc$V1+sex$V1+age$V1+edyears$V1+bmi$V1+pc

1$V1+pc2$V1+pc3$V1+pc4$V1+pc5$V1+pc6$V1+pc7$V1+pc8$V1+pc9$V1+pc10$V1,fami

ly=gaussian))

pval <- result$coefficients[2,4]

b=result$coefficients[2,1]

se=result$coefficients[2,2]

Output[1,n]<-b

Output[2,n]<-se

Output[3,n]<-pval

}

write.csv(Output, file="Output_Admixture_sbp10_PC.csv")

#################

#################

#Association analysis

#from Jacklyn Quinlan et al

#Sept, 2015

#################

#################

geno1<-read.table(file="27559_geno_MAF0.01_HWE3_Miss0.05.tsv",

header=F,sep="\t", na.strings="NA")

phenotype<-read.table(file="157_sbp10.tsv", header=F,sep="\t",

na.strings="NA")

GlobalMeanAncestry<-read.table(file="157_GA.tsv", header=F,sep="\t",

na.strings="NA")

sex<-read.table(file="157_sex.tsv", header=F,sep="\t", na.strings="NA")

age<-read.table(file="157_age.tsv", header=F,sep="\t", na.strings="NA")

bmi<-read.table(file="157_bmi.tsv", header=F,sep="\t", na.strings="NA")

edyears<-read.table(file="157_edyears.tsv", header=F,sep="\t",

na.strings="NA")

pc1<-read.table(file="157_pcair1.tsv", header=F,sep="\t",

na.strings="NA")

pc2<-read.table(file="157_pcair2.tsv", header=F,sep="\t",

na.strings="NA")

pc3<-read.table(file="157_pcair3.tsv", header=F,sep="\t",

na.strings="NA")

pc4<-read.table(file="157_pcair4.tsv", header=F,sep="\t",

na.strings="NA")

pc5<-read.table(file="157_pcair5.tsv", header=F,sep="\t",

na.strings="NA")

pc6<-read.table(file="157_pcair6.tsv", header=F,sep="\t",

na.strings="NA")

pc7<-read.table(file="157_pcair7.tsv", header=F,sep="\t",

na.strings="NA")

pc8<-read.table(file="157_pcair8.tsv", header=F,sep="\t",

na.strings="NA")

pc9<-read.table(file="157_pcair9.tsv", header=F,sep="\t",

na.strings="NA")

pc10<-read.table(file="157_pcair10.tsv", header=F,sep="\t",

na.strings="NA")

headers<-read.csv(file="header.csv",sep=",")

Output<-matrix(nrow=3,ncol=ncol(headers))

colnames(Output)<-colnames(headers)

rownames(Output) <- c("b", "se", "pval")

#phenotype is the vector of phenotypes

#geno is the vector of genotypes at a marker

for (n in (1: 27559)){

geno<-c(geno1[[n]])

y<-c(phenotype)

globalanc<-c(GlobalMeanAncestry)

result <-

summary(glm(y$V1~geno+globalanc$V1+sex$V1+age$V1+edyears$V1+bmi$V1+pc1$V1

+pc2$V1+pc3$V1+pc4$V1+pc5$V1+pc6$V1+pc7$V1+pc8$V1+pc9$V1+pc10$V1,family=g

aussian))

pval <- result$coefficients[2,4]

b=result$coefficients[2,1]

se=result$coefficients[2,2]

Output[1,n]<-b

Output[2,n]<-se

Output[3,n]<-pval

}

write.csv(Output, file="Output_GWAS_sbp10_PC.csv")

#################

#################

#Joint ancestry and association analysis Model 1

#Implementation of the BMIX test

#adapted from Daniel Shriner, July 28,2011 by Jacklyn Quinlan, et al.

#Sept, 2015

#################

#################

#################

#Data is read in:

#phenotype is the vector of phenotypes.

#localanc is the vector of local ancestry estimates at a marker.

#globalanc is the vector of global ancestry estimates at a marker.

#geno is the vector of recoded genotypes at a marker.

#################

localanc1<-

read.table(file="27559_LAMPancestry_estimates_MAF0.01_HWE3_Miss0.05_code0

_1_2.tsv", header=F,sep="\t", na.strings="NA")

geno1<-

read.table(file="27559_geno_MAF0.01_HWE3_Miss0.05.tsv",header=F,sep="\t",

na.strings="NA")

phenotype<-read.table(file="157_sbp10.tsv", header=F,sep="\t",

na.strings="NA")

GlobalMeanAncestry<-read.table(file="157_GA.tsv", header=F,sep="\t",

na.strings="NA")

sex<-read.table(file="157_sex.tsv", header=F,sep="\t", na.strings="NA")

age<-read.table(file="157_age.tsv", header=F,sep="\t", na.strings="NA")

bmi<-read.table(file="157_bmi.tsv", header=F,sep="\t", na.strings="NA")

edyears<-read.table(file="157_edyears.tsv", header=F,sep="\t",

na.strings="NA")

pc1<-read.table(file="157_pcair1.tsv", header=F,sep="\t",

na.strings="NA")

pc2<-read.table(file="157_pcair2.tsv", header=F,sep="\t",

na.strings="NA")

pc3<-read.table(file="157_pcair3.tsv", header=F,sep="\t",

na.strings="NA")

pc4<-read.table(file="157_pcair4.tsv", header=F,sep="\t",

na.strings="NA")

pc5<-read.table(file="157_pcair5.tsv", header=F,sep="\t",

na.strings="NA")

pc6<-read.table(file="157_pcair6.tsv", header=F,sep="\t",

na.strings="NA")

pc7<-read.table(file="157_pcair7.tsv", header=F,sep="\t",

na.strings="NA")

pc8<-read.table(file="157_pcair8.tsv", header=F,sep="\t",

na.strings="NA")

pc9<-read.table(file="157_pcair9.tsv", header=F,sep="\t",

na.strings="NA")

pc10<-read.table(file="157_pcair10.tsv", header=F,sep="\t",

na.strings="NA")

headers<-read.csv(file="header.csv",sep=",")

Output<-matrix(nrow=6,ncol=ncol(headers))

colnames(Output)<-colnames(headers)

rownames(Output) <- c("pooled.beta", "pooled.se", "pooled.pval",

"admixture_posterior", "GWAS_posterior", "joint_posterior")

#################

#Each site is now tested for association.

#################

for (n in 1:ncol(headers)){

localanc<-c(localanc1[[n]])

geno<-c(geno1[[n]])

y<-c(phenotype)

globalanc<-c(GlobalMeanAncestry)

posterior <- function(x,prior,lambda)

{(dchisq(x,1,lambda)*prior)/((dchisq(x,1,lambda)*prior)+(dchisq(x,1,0)*(1

-prior)))}

admixture_burden <- 78.82 #for the Tallahassee data

association_burden <- 27559 #for the Tallahassee data

#################

#In the following section the admixture posterior is calculated using both #local and global ancestry while taking into account the number of tests.

#The admixture posterior will later be used as a prior for the joint #association test.

#################

result <-

summary(glm(y$V1~localanc+globalanc$V1+sex$V1+age$V1+bmi$V1+edyears$V1+pc

1$V1+pc2$V1+pc3$V1+pc4$V1+pc5$V1+pc6$V1+pc7$V1+pc8$V1+pc9$V1+pc10$V1,fami

ly=gaussian))

admixture_p <- result$coefficients[2,4]

admixture_lambda <- (qnorm(1-0.05/admixture_burden/2)+qnorm(0.8))^2

admixture_prior <- 1/admixture_burden

admixture_test <- qchisq(admixture_p,1,0,lower.tail=FALSE)

admixture_posterior <-

posterior(x=admixture_test,prior=admixture_prior,lambda=admixture_lambda)

#Stratified association testing is performed where individual are grouped #based on local ancestry at that site.

group0<-which(localanc==0)

if ((is.element(0,geno[group0]) & is.element(1,geno[group0])) |

(is.element(1,geno[group0]) & is.element(2,geno[group0])) |

(is.element(0,geno[group0]) & is.element(2,geno[group0])))

{

result <-summary(glm(y$V1[group0]~geno[group0]+globalanc$V1[group0]+sex$V1[group0]

+age$V1[group0]+edyears$V1[group0]+bmi$V1[group0]+pc1$V1[group0]+pc2$V1[g

roup0]+pc3$V1[group0]+pc4$V1[group0]+pc5$V1[group0]+pc6$V1[group0]+pc7$V1

[group0]+pc8$V1[group0]+pc9$V1[group0]+pc10$V1[group0],family=gaussian))

if (nrow(result$coefficients)==17){(tmp1 <-

result$coefficients[2,1]) & (tmp2 <- result$coefficients[2,2])

}else{

(tmp1<-NA) & (tmp2<-NA)}

} else {

tmp1 <- NA

tmp2 <- NA

}

group1 <- which(localanc==1)

if ((is.element(0,geno[group1]) & is.element(1,geno[group1])) |

(is.element(1,geno[group1]) & is.element(2,geno[group1])) |

(is.element(0,geno[group1]) & is.element(2,geno[group1]))) {

result <-

summary(glm(y$V1[group1]~geno[group1]+globalanc$V1[group1]+sex$V1[group1]

+age$V1[group1]+edyears$V1[group1]+bmi$V1[group1]+pc1$V1[group1]+pc2$V1[g

roup1]+pc3$V1[group1]+pc4$V1[group1]+pc5$V1[group1]+pc6$V1[group1]+pc7$V1

[group1]+pc8$V1[group1]+pc9$V1[group1]+pc10$V1[group1],family=gaussian))

if (nrow(result$coefficients)==17){(tmp3 <-

result$coefficients[2,1]) & (tmp4 <- result$coefficients[2,2])

}else{

(tmp3<-NA) & (tmp4<-NA)}

} else {

tmp3 <- NA

tmp4 <- NA

}

group2 <- which(localanc==2)

if ((is.element(0,geno[group2]) & is.element(1,geno[group2])) |

(is.element(1,geno[group2]) & is.element(2,geno[group2])) |

(is.element(0,geno[group2]) & is.element(2,geno[group2]))) {

result <-

summary(glm(y$V1[group2]~geno[group2]+globalanc$V1[group2]+sex$V1[group2]

+age$V1[group2]+edyears$V1[group2]+bmi$V1[group2]+pc1$V1[group2]+pc2$V1[g

roup2]+pc3$V1[group2]+pc4$V1[group2]+pc5$V1[group2]+pc6$V1[group2]+pc7$V1

[group2]+pc8$V1[group2]+pc9$V1[group2]+pc10$V1[group2],family=gaussian))

if (nrow(result$coefficients)==17){(tmp5 <-

result$coefficients[2,1]) & (tmp6 <- result$coefficients[2,2])

}else{

(tmp5<-NA) & (tmp6<-NA)}

} else {

tmp5 <- NA

tmp6 <- NA

}

#################

#Inverse variance-weighted fixed effects are then corrected for and the #p-values are pooled.

#################

pooled.pval <- NA

if (!any(is.na(c(tmp1,tmp2,tmp3,tmp4,tmp5,tmp6)))) {

pooled.beta <-

((tmp1/(tmp2^2))+(tmp3/(tmp4^2))+(tmp5/(tmp6^2)))/(1/(tmp2^2)+1/(tmp4^2)+

1/(tmp6^2))

pooled.se <- sqrt(1/(1/(tmp2^2)+1/(tmp4^2)+1/(tmp6^2)))

pooled.pval <- 2*(1-pnorm(abs(pooled.beta/pooled.se)))

}

if (!is.na(tmp1) && !is.na(tmp2) && !is.na(tmp3) && !is.na(tmp4) &&

is.na(tmp5) && is.na(tmp6)) {

pooled.beta <-

((tmp1/(tmp2^2))+(tmp3/(tmp4^2)))/(1/(tmp2^2)+1/(tmp4^2))

pooled.se <- sqrt(1/(1/(tmp2^2)+1/(tmp4^2)))

pooled.pval <- 2*(1-pnorm(abs(pooled.beta/pooled.se)))

}

if (!is.na(tmp1) && !is.na(tmp2) && is.na(tmp3) && is.na(tmp4) &&

!is.na(tmp5) && !is.na(tmp6)) {

pooled.beta <-

((tmp1/(tmp2^2))+(tmp5/(tmp6^2)))/(1/(tmp2^2)+1/(tmp6^2))

pooled.se <- sqrt(1/(1/(tmp2^2)+1/(tmp6^2)))

pooled.pval <- 2*(1-pnorm(abs(pooled.beta/pooled.se)))

}

if (is.na(tmp1) && is.na(tmp2) && !is.na(tmp3) && !is.na(tmp4) &&

!is.na(tmp5) && !is.na(tmp6)) {

pooled.beta <-

((tmp3/(tmp4^2))+(tmp5/(tmp6^2)))/(1/(tmp4^2)+1/(tmp6^2))

pooled.se <- sqrt(1/(1/(tmp4^2)+1/(tmp6^2)))

pooled.pval <- 2*(1-pnorm(abs(pooled.beta/pooled.se)))

}

if (!is.na(tmp1) && !is.na(tmp2) && is.na(tmp3) && is.na(tmp4) &&

is.na(tmp5) && is.na(tmp6)) {

pooled.beta <- tmp1

pooled.se <- tmp2

pooled.pval <- 2*(1-pnorm(abs(pooled.beta/pooled.se)))

}

if (is.na(tmp1) && is.na(tmp2) && !is.na(tmp3) && !is.na(tmp4) &&

is.na(tmp5) && is.na(tmp6)) {

pooled.beta <- tmp3

pooled.se <- tmp4

pooled.pval <- 2*(1-pnorm(abs(pooled.beta/pooled.se)))

}

if (is.na(tmp1) && is.na(tmp2) && is.na(tmp3) && is.na(tmp4) &&

!is.na(tmp5) && !is.na(tmp6)) {

pooled.beta <- tmp5

pooled.se <- tmp6

pooled.pval <- 2*(1-pnorm(abs(pooled.beta/pooled.se)))

}

#################

#The joint posterior is now generated based on the pooled p-value with the #admixture posterior being used as a prior for the joint test.

#################

if (!is.na(pooled.pval))

{

association_lambda <- (qnorm(1-

0.05/association_burden/2)+qnorm(0.8))^2

association_test <- qchisq(pooled.pval,1,0,lower.tail=FALSE)

joint_posterior <- posterior(x=association_test,

prior=admixture_posterior, lambda=association_lambda)

} else {

joint_posterior <- NA

}

association_prior <- 1/association_burden

GWAS_posterior<- posterior(x=association_test, prior=association_prior,

lambda=association_lambda)

Output[1,n]<-pooled.beta

Output[2,n]<-pooled.se

Output[3,n]<-pooled.pval

Output[4,n]<-admixture_posterior

Output[5,n]<-GWAS_posterior

Output[6,n]<-joint_posterior

}

write.csv(Output, file="SNP_Model1_Output_SBP_edyears_PCs10.csv")

#################

#################

#Joint ancestry and association analysis Model 2

#Implementation of the BMIX test

#adapted from Daniel Shriner, July 28,2011 by Jacklyn Quinlan, et al.

#Sept, 2015

#################

#################

#################

#Data is read in:

#phenotype is the vector of phenotypes

#localanc is the vector of local ancestry estimates at a marker

#globalanc is the vector of global ancestry estimates at a marker

#geno is the vector of recoded genotypes at a marker

#################

localanc1<-

read.table(file="27559_LAMPancestry_estimates_MAF0.01,_HWE3_MISS0.05_code

0_1_2.tsv", header=F,sep="\t", na.strings="NA")

geno1<-

read.table(file="27559_geno_MAF0.01_HWE3_Miss0.05.tsv",header=F,sep="\t",

na.strings="NA")

phenotype<-read.table(file="157_sbp10.tsv", header=F,sep="\t",

na.strings="NA")

GlobalMeanAncestry<-read.table(file="157_GA.tsv", header=F,sep="\t",

na.strings="NA")

sex<-read.table(file="157_sex.tsv", header=F,sep="\t", na.strings="NA")

age<-read.table(file="157_age.tsv", header=F,sep="\t", na.strings="NA")

bmi<-read.table(file="157_bmi.tsv", header=F,sep="\t", na.strings="NA")

edyears<-read.table(file="157_edyears.tsv", header=F,sep="\t",

na.strings="NA")

ut<-read.table(file="157_utother.tsv", header=F,sep="\t",

na.strings="NA")

pc1<-read.table(file="157_pcair1.tsv", header=F,sep="\t",

na.strings="NA")

pc2<-read.table(file="157_pcair2.tsv", header=F,sep="\t",

na.strings="NA")

pc3<-read.table(file="157_pcair3.tsv", header=F,sep="\t",

na.strings="NA")

pc4<-read.table(file="157_pcair4.tsv", header=F,sep="\t",

na.strings="NA")

pc5<-read.table(file="157_pcair5.tsv", header=F,sep="\t",

na.strings="NA")

pc6<-read.table(file="157_pcair6.tsv", header=F,sep="\t",

na.strings="NA")

pc7<-read.table(file="157_pcair7.tsv", header=F,sep="\t",

na.strings="NA")

pc8<-read.table(file="157_pcair8.tsv", header=F,sep="\t",

na.strings="NA")

pc9<-read.table(file="157_pcair9.tsv", header=F,sep="\t",

na.strings="NA")

pc10<-read.table(file="157_pcair10.tsv", header=F,sep="\t",

na.strings="NA")

headers<-read.csv(file="header.csv",sep=",")

Output<-matrix(nrow=6,ncol=ncol(headers))

colnames(Output)<-colnames(headers)

rownames(Output) <- c("pooled.beta", "pooled.se", "pooled.pval",

"admixture_posterior", "GWAS_posterior", "joint_posterior")

#################

#Each site is now tested for association.

#################

for (n in 1:27559) {

localanc<-c(localanc1[[n]])

geno<-c(geno1[[n]])

utother<-c(ut)

y<-c(phenotype)

globalanc<-c(GlobalMeanAncestry)

posterior <- function(x,prior,lambda)

{(dchisq(x,1,lambda)*prior)/((dchisq(x,1,lambda)*prior)+(dchisq(x,1,0)*(1

-prior)))}

admixture_burden <- 78.82 #for the Tallahassee data

association_burden <- 27559 #for the Tallahassee data

#################

#In the following section the admixture posterior is calculated using both #local and global ancestry while taking into account the number of tests.

#The admixture posterior will later be used as a prior for the joint. #association test.

#################

result <-

summary(glm(y$V1~localanc+globalanc$V1+utother$V1+sex$V1+age$V1+bmi$V1+ed

years$V1+pc1$V1+pc2$V1+pc3$V1+pc4$V1+pc5$V1+pc6$V1+pc7$V1+pc8$V1+pc9$V1+p

c10$V1,family=gaussian))

admixture_p <- result$coefficients[2,4]

admixture_lambda <- (qnorm(1-0.05/admixture_burden/2)+qnorm(0.8))^2

admixture_prior <- 1/admixture_burden

admixture_test <- qchisq(admixture_p,1,0,lower.tail=FALSE)

admixture_posterior <-

posterior(x=admixture_test,prior=admixture_prior,lambda=admixture_lambda)

#################

#Stratified association testing is performed where individual are grouped #based on local ancestry at that site.

#################

group0<-which(localanc==0)

if ((is.element(0,geno[group0]) & is.element(1,geno[group0])) |

(is.element(1,geno[group0]) & is.element(2,geno[group0])) |

(is.element(0,geno[group0]) & is.element(2,geno[group0])))

{

result <-

summary(glm(y$V1[group0]~geno[group0]+globalanc$V1[group0]+utother$V1[gro

up0]+sex$V1[group0]+age$V1[group0]+bmi$V1[group0]+edyears$V1[group0]+pc1$

V1[group0]+pc2$V1[group0]+pc3$V1[group0]+pc4$V1[group0]+pc5$V1[group0]+pc

6$V1[group0]+pc7$V1[group0]+pc8$V1[group0]+pc9$V1[group0]+pc10$V1[group0]

,family=gaussian))

if (nrow(result$coefficients)==18){(tmp1 <-

result$coefficients[2,1]) & (tmp2 <- result$coefficients[2,2])

}else{

(tmp1<-NA) & (tmp2<-NA)}

} else {

tmp1 <- NA

tmp2 <- NA

}

group1 <- which(localanc==1)

if ((is.element(0,geno[group1]) & is.element(1,geno[group1])) |

(is.element(1,geno[group1]) & is.element(2,geno[group1])) |

(is.element(0,geno[group1]) & is.element(2,geno[group1]))) {

result <-

summary(glm(y$V1[group1]~geno[group1]+globalanc$V1[group1]+utother$V1[gro

up1]+sex$V1[group1]+age$V1[group1]+bmi$V1[group1]+edyears$V1[group1]+pc1$

V1[group1]+pc2$V1[group1]+pc3$V1[group1]+pc4$V1[group1]+pc5$V1[group1]+pc

6$V1[group1]+pc7$V1[group1]+pc8$V1[group1]+pc9$V1[group1]+pc10$V1[group1]

,family=gaussian))

if (nrow(result$coefficients)==18){(tmp3 <-

result$coefficients[2,1]) & (tmp4 <- result$coefficients[2,2])

}else{

(tmp3<-NA) & (tmp4<-NA)}

} else {

tmp3 <- NA

tmp4 <- NA

}

group2 <- which(localanc==2)

if ((is.element(0,geno[group2]) & is.element(1,geno[group2])) |

(is.element(1,geno[group2]) & is.element(2,geno[group2])) |

(is.element(0,geno[group2]) & is.element(2,geno[group2]))) {

result <-

summary(glm(y$V1[group2]~geno[group2]+globalanc$V1[group2]+utother$V1[gro

up2]+sex$V1[group2]+age$V1[group2]+bmi$V1[group2]+edyears$V1[group2]+pc1$

V1[group2]+pc2$V1[group2]+pc3$V1[group2]+pc4$V1[group2]+pc5$V1[group2]+pc

6$V1[group2]+pc7$V1[group2]+pc8$V1[group2]+pc9$V1[group2]+pc10$V1[group2]

,family=gaussian))

if (nrow(result$coefficients)==18){(tmp5 <-

result$coefficients[2,1]) & (tmp6 <- result$coefficients[2,2])

}else{

(tmp5<-NA) & (tmp6<-NA)}

} else {

tmp5 <- NA

tmp6 <- NA

}

#################

#Inverse variance-weighted fixed effects are then corrected for and the #p-values are pooled.

#################

pooled.pval <- NA

if (!any(is.na(c(tmp1,tmp2,tmp3,tmp4,tmp5,tmp6)))) {

pooled.beta <-

((tmp1/(tmp2^2))+(tmp3/(tmp4^2))+(tmp5/(tmp6^2)))/(1/(tmp2^2)+1/(tmp4^2)+

1/(tmp6^2))

pooled.se <- sqrt(1/(1/(tmp2^2)+1/(tmp4^2)+1/(tmp6^2)))

pooled.pval <- 2*(1-pnorm(abs(pooled.beta/pooled.se)))

}

if (!is.na(tmp1) && !is.na(tmp2) && !is.na(tmp3) && !is.na(tmp4) &&

is.na(tmp5) && is.na(tmp6)) {

pooled.beta <-

((tmp1/(tmp2^2))+(tmp3/(tmp4^2)))/(1/(tmp2^2)+1/(tmp4^2))

pooled.se <- sqrt(1/(1/(tmp2^2)+1/(tmp4^2)))

pooled.pval <- 2*(1-pnorm(abs(pooled.beta/pooled.se)))

}

if (!is.na(tmp1) && !is.na(tmp2) && is.na(tmp3) && is.na(tmp4) &&

!is.na(tmp5) && !is.na(tmp6)) {

pooled.beta <-

((tmp1/(tmp2^2))+(tmp5/(tmp6^2)))/(1/(tmp2^2)+1/(tmp6^2))

pooled.se <- sqrt(1/(1/(tmp2^2)+1/(tmp6^2)))

pooled.pval <- 2*(1-pnorm(abs(pooled.beta/pooled.se)))

}

if (is.na(tmp1) && is.na(tmp2) && !is.na(tmp3) && !is.na(tmp4) &&

!is.na(tmp5) && !is.na(tmp6)) {

pooled.beta <-

((tmp3/(tmp4^2))+(tmp5/(tmp6^2)))/(1/(tmp4^2)+1/(tmp6^2))

pooled.se <- sqrt(1/(1/(tmp4^2)+1/(tmp6^2)))

pooled.pval <- 2*(1-pnorm(abs(pooled.beta/pooled.se)))

}

if (!is.na(tmp1) && !is.na(tmp2) && is.na(tmp3) && is.na(tmp4) &&

is.na(tmp5) && is.na(tmp6)) {

pooled.beta <- tmp1

pooled.se <- tmp2

pooled.pval <- 2*(1-pnorm(abs(pooled.beta/pooled.se)))

}

if (is.na(tmp1) && is.na(tmp2) && !is.na(tmp3) && !is.na(tmp4) &&

is.na(tmp5) && is.na(tmp6)) {

pooled.beta <- tmp3

pooled.se <- tmp4

pooled.pval <- 2*(1-pnorm(abs(pooled.beta/pooled.se)))

}

if (is.na(tmp1) && is.na(tmp2) && is.na(tmp3) && is.na(tmp4) &&

!is.na(tmp5) && !is.na(tmp6)) {

pooled.beta <- tmp5

pooled.se <- tmp6

pooled.pval <- 2*(1-pnorm(abs(pooled.beta/pooled.se)))

}

#################

#The joint posterior is now generated based on the pooled p-value with the #admixture posterior being used as a prior for the joint test.

#################

if (!is.na(pooled.pval))

{

association_lambda <- (qnorm(1-

0.05/association_burden/2)+qnorm(0.8))^2

association_test <- qchisq(pooled.pval,1,0,lower.tail=FALSE)

joint_posterior <- posterior(x=association_test,

prior=admixture_posterior, lambda=association_lambda)

} else {

joint_posterior <- NA

}

association_prior <- 1/association_burden

GWAS_posterior<- posterior(x=association_test, prior=association_prior,

lambda=association_lambda)

Output[1,n]<-pooled.beta

Output[2,n]<-pooled.se

Output[3,n]<-pooled.pval

Output[4,n]<-admixture_posterior

Output[5,n]<-GWAS_posterior

Output[6,n]<-joint_posterior

}

write.csv(Output, file="SNP_Model2_utother_SBP_edyears_10PCs.csv")

#################

#################

#Joint ancestry and association analysis Model 3

#Implementation of the BMIX test

#adapted from Daniel Shriner, July 28,2011 by Jacklyn Quinlan, et al.

#Sept, 2015

#################

#################

#################

#Data is read in:

#phenotype is the vector of phenotypes.

#localanc is the vector of local ancestry estimates at a marker.

#globalanc is the vector of global ancestry estimates at a marker.

#geno is the vector of recoded genotypes at a marker.

#################

localanc1<-

read.table(file="27559_LAMPancestry_estimates_MAF0.01,_HWE3_MISS0.05_code

0_1_2.tsv", header=F,sep="\t", na.strings="NA")

geno1<-

read.table(file="27559_geno_MAF0.01_HWE3_Miss0.05.tsv",header=F,sep="\t",

na.strings="NA")

phenotype<-read.table(file="157_sbp10.tsv", header=F,sep="\t",

na.strings="NA")

GlobalMeanAncestry<-read.table(file="157_GA.tsv", header=F,sep="\t",

na.strings="NA")

ut<-read.table(file="157_utother_high_low.tsv", header=F,sep="\t",

na.strings="NA")

sex<-read.table(file="157_sex.tsv", header=F,sep="\t", na.strings="NA")

age<-read.table(file="157_age.tsv", header=F,sep="\t", na.strings="NA")

bmi<-read.table(file="157_bmi.tsv", header=F,sep="\t", na.strings="NA")

edyears<-read.table(file="157_edyears.tsv", header=F,sep="\t",

na.strings="NA")

pc1<-read.table(file="157_pcair1.tsv", header=F,sep="\t",

na.strings="NA")

pc2<-read.table(file="157_pcair2.tsv", header=F,sep="\t",

na.strings="NA")

pc3<-read.table(file="157_pcair3.tsv", header=F,sep="\t",

na.strings="NA")

pc4<-read.table(file="157_pcair4.tsv", header=F,sep="\t",

na.strings="NA")

pc5<-read.table(file="157_pcair5.tsv", header=F,sep="\t",

na.strings="NA")

pc6<-read.table(file="157_pcair6.tsv", header=F,sep="\t",

na.strings="NA")

pc7<-read.table(file="157_pcair7.tsv", header=F,sep="\t",

na.strings="NA")

pc8<-read.table(file="157_pcair8.tsv", header=F,sep="\t",

na.strings="NA")

pc9<-read.table(file="157_pcair9.tsv", header=F,sep="\t",

na.strings="NA")

pc10<-read.table(file="157_pcair10.tsv", header=F,sep="\t",

na.strings="NA")

headers<-read.csv(file="header.csv",sep=",")

Output<-matrix(nrow=6,ncol=ncol(headers))

colnames(Output)<-colnames(headers)

rownames(Output) <- c("pooled.beta", "pooled.se", "pooled.pval",

"admixture_posterior", "GWAS_posterior", "joint_posterior")

#################

#Each site is now tested for association.

#################

for (n in 1:ncol(headers)) {

localanc<-c(localanc1[[n]])

geno<-c(geno1[[n]])

utother<-c(ut)

y<-c(phenotype)

globalanc<-c(GlobalMeanAncestry)

posterior <- function(x,prior,lambda)

{(dchisq(x,1,lambda)*prior)/((dchisq(x,1,lambda)*prior)+(dchisq(x,1,0)*(1

-prior)))}

admixture_burden <- 78.82166 #for the Tallahassee data

association_burden <- 18324 #for MAF10% out of 27559 Tallahassee SNPs

#################

#In the following section the admixture posterior is calculated using both #local and global ancestry while taking into account the number of tests.

#The admixture posterior will later be used as a prior for the joint. #association test.

#################

result <-

summary(glm(y$V1~localanc+globalanc$V1+utother$V1+sex$V1+age$V1+bmi$V1+edyears$V1+utother$V1*localanc+pc1$V1+pc2$V1+pc3$V1+pc4$V1+pc5$V1+pc6$V1+pc7$V1+pc8$V1+pc9$V1+pc10$V1,family=gaussian))

admixture_p <- result$coefficients[19,4]

admixture_lambda <- (qnorm(1-0.05/admixture_burden/2)+qnorm(0.8))^2

admixture_prior <- 1/admixture_burden

admixture_test <- qchisq(admixture_p,1,0,lower.tail=FALSE)

admixture_posterior <-

posterior(x=admixture_test,prior=admixture_prior,lambda=admixture_lambda)

#################

#Stratified association testing is performed where individual are grouped #based on local ancestry at that site.

#################

group0<-which(localanc==0)

if ((is.element(0,geno[group0]) & is.element(1,geno[group0])) |

(is.element(1,geno[group0]) & is.element(2,geno[group0])) |

(is.element(0,geno[group0]) & is.element(2,geno[group0])))

{

result <-

summary(glm(y$V1[group0]~geno[group0]+globalanc$V1[group0]+utother$V1[group0]+sex$V1[group0]+age$V1[group0]+bmi$V1[group0]+edyears$V1[group0]+utother$V1[group0]*geno[group0]+pc1$V1[group0]+pc2$V1[group0]+pc3$V1[group0]+pc4$V1[group0]+pc5$V1[group0]+pc6$V1[group0]+pc7$V1[group0]+pc8$V1[group0]+pc

9$V1[group0]+pc10$V1[group0],family=gaussian))

if (nrow(result$coefficients)==19){(tmp1 <-

result$coefficients[19,1]) & (tmp2 <- result$coefficients[19,2])

}else{

(tmp1<-NA) & (tmp2<-NA)}

} else {

tmp1 <- NA

tmp2 <- NA

}

group1 <- which(localanc==1)

if ((is.element(0,geno[group1]) & is.element(1,geno[group1])) |

(is.element(1,geno[group1]) & is.element(2,geno[group1])) |

(is.element(0,geno[group1]) & is.element(2,geno[group1]))) {

result <-

summary(glm(y$V1[group1]~geno[group1]+globalanc$V1[group1]+utother$V1[group1]+sex$V1[group1]+age$V1[group1]+bmi$V1[group1]+edyears$V1[group1]+utother$V1[group1]*geno[group1]+pc1$V1[group1]+pc2$V1[group1]+pc3$V1[group1]+pc4$V1[group1]+pc5$V1[group1]+pc6$V1[group1]+pc7$V1[group1]+pc8$V1[group1]+pc

9$V1[group1]+pc10$V1[group1],family=gaussian))

if (nrow(result$coefficients)==19){(tmp3 <-

result$coefficients[19,1]) & (tmp4 <- result$coefficients[19,2])

}else{

(tmp3<-NA) & (tmp4<-NA)}

} else {

tmp3 <- NA

tmp4 <- NA

}

group2 <- which(localanc==2)

if ((is.element(0,geno[group2]) & is.element(1,geno[group2])) |

(is.element(1,geno[group2]) & is.element(2,geno[group2])) |

(is.element(0,geno[group2]) & is.element(2,geno[group2]))) {

result <-

summary(glm(y$V1[group2]~geno[group2]+globalanc$V1[group2]+utother$V1[group2]+sex$V1[group2]+age$V1[group2]+bmi$V1[group2]+edyears$V1[group2]+utother$V1[group2]*geno[group2]+pc1$V1[group2]+pc2$V1[group2]+pc3$V1[group2]+pc4$V1[group2]+pc5$V1[group2]+pc6$V1[group2]+pc7$V1[group2]+pc8$V1[group2]+pc

9$V1[group2]+pc10$V1[group2],family=gaussian))

if (nrow(result$coefficients)==19){(tmp5 <-

result$coefficients[19,1]) & (tmp6 <- result$coefficients[19,2])

}else{

(tmp5<-NA) & (tmp6<-NA)}

} else {

tmp5 <- NA

tmp6 <- NA

}

#################

#Inverse variance-weighted fixed effects are then corrected for and the #p-values are pooled.

#################

pooled.pval <- NA

if (!any(is.na(c(tmp1,tmp2,tmp3,tmp4,tmp5,tmp6)))) {

pooled.beta <-

((tmp1/(tmp2^2))+(tmp3/(tmp4^2))+(tmp5/(tmp6^2)))/(1/(tmp2^2)+1/(tmp4^2)+

1/(tmp6^2))

pooled.se <- sqrt(1/(1/(tmp2^2)+1/(tmp4^2)+1/(tmp6^2)))

pooled.pval <- 2*(1-pnorm(abs(pooled.beta/pooled.se)))

}

if (!is.na(tmp1) && !is.na(tmp2) && !is.na(tmp3) && !is.na(tmp4) &&

is.na(tmp5) && is.na(tmp6)) {

pooled.beta <-

((tmp1/(tmp2^2))+(tmp3/(tmp4^2)))/(1/(tmp2^2)+1/(tmp4^2))

pooled.se <- sqrt(1/(1/(tmp2^2)+1/(tmp4^2)))

pooled.pval <- 2*(1-pnorm(abs(pooled.beta/pooled.se)))

}

if (!is.na(tmp1) && !is.na(tmp2) && is.na(tmp3) && is.na(tmp4) &&

!is.na(tmp5) && !is.na(tmp6)) {

pooled.beta <-

((tmp1/(tmp2^2))+(tmp5/(tmp6^2)))/(1/(tmp2^2)+1/(tmp6^2))

pooled.se <- sqrt(1/(1/(tmp2^2)+1/(tmp6^2)))

pooled.pval <- 2*(1-pnorm(abs(pooled.beta/pooled.se)))

}

if (is.na(tmp1) && is.na(tmp2) && !is.na(tmp3) && !is.na(tmp4) &&

!is.na(tmp5) && !is.na(tmp6)) {

pooled.beta <-

((tmp3/(tmp4^2))+(tmp5/(tmp6^2)))/(1/(tmp4^2)+1/(tmp6^2))

pooled.se <- sqrt(1/(1/(tmp4^2)+1/(tmp6^2)))

pooled.pval <- 2*(1-pnorm(abs(pooled.beta/pooled.se)))

}

if (!is.na(tmp1) && !is.na(tmp2) && is.na(tmp3) && is.na(tmp4) &&

is.na(tmp5) && is.na(tmp6)) {

pooled.beta <- tmp1

pooled.se <- tmp2

pooled.pval <- 2*(1-pnorm(abs(pooled.beta/pooled.se)))

}

if (is.na(tmp1) && is.na(tmp2) && !is.na(tmp3) && !is.na(tmp4) &&

is.na(tmp5) && is.na(tmp6)) {

pooled.beta <- tmp3

pooled.se <- tmp4

pooled.pval <- 2*(1-pnorm(abs(pooled.beta/pooled.se)))

}

if (is.na(tmp1) && is.na(tmp2) && is.na(tmp3) && is.na(tmp4) &&

!is.na(tmp5) && !is.na(tmp6)) {

pooled.beta <- tmp5

pooled.se <- tmp6

pooled.pval <- 2*(1-pnorm(abs(pooled.beta/pooled.se)))

}

#################

#The joint posterior is now generated based on the pooled p-value with the #admixture posterior being used as a prior for the joint test.

#################

if (!is.na(pooled.pval))

{

association_lambda <- (qnorm(1-

0.05/association_burden/2)+qnorm(0.8))^2

association_test <- qchisq(pooled.pval,1,0,lower.tail=FALSE)

joint_posterior <- posterior(x=association_test,

prior=admixture_posterior, lambda=association_lambda)

} else {

joint_posterior <- NA

}

association_prior <- 1/association_burden

GWAS_posterior<- posterior(x=association_test, prior=association_prior,

lambda=association_lambda)

Output[1,n]<-pooled.beta

Output[2,n]<-pooled.se

Output[3,n]<-pooled.pval

Output[4,n]<-admixture_posterior

Output[5,n]<-GWAS_posterior

Output[6,n]<-joint_posterior

}

write.csv(Output, file="Interaction_Int_effect_utother_high_low_Output_SBP_PCs10.csv")

#################

#################

#Simulation ancestry and association analysis Model 1

#Implementation of the BMIX test

#adapted from Daniel Shriner, July 28,2011 by Jacklyn Quinlan, et al.

#Sept, 2015 and above association code

#################

#################

BMIXsimed<-function(localanc1, geno1, phenotypeIN, GlobalMeanAncestryIN, sexIN, ageIN, bmiIN, edyearsIN, PCair1, PCair2, PCair3, PCair4, PCair5, PCair6, PCair7, PCair8, PCair9, PCair10, headers, n.sims) {

rownumber<-1:157

pc1<-PCair1

pc2<-PCair2

pc3<-PCair3

pc4<-PCair4

pc5<-PCair5

pc6<-PCair6

pc7<-PCair7

pc8<-PCair8

pc9<-PCair9

pc10<-PCair10

#################

#Output matrices are defined by the number of sites tested and #simulations run

#################

simoutputadmix<-matrix(nrow=ncol(headers),ncol=n.sims)

simoutputGWAS<-matrix(nrow=ncol(headers),ncol=n.sims)

simoutputjoint<-matrix(nrow=ncol(headers),ncol=n.sims)

simoutput<-list(simoutputadmix, simoutputGWAS, simoutputjoint)

for (k in 1:n.sims){

#################

#Row numbers are shuffled each run of the simulation

#################

newrowindex<-sample(rownumber)

#################

#Ancestry remains linked to genotype, while phenotype, sex, age, bmi, and #edyears are shuffled based on row numbers

#################

phenotype<-as.data.frame(as.matrix(phenotypeIN[newrowindex,1]))

GlobalMeanAncestry<-as.data.frame(as.matrix(GlobalMeanAncestryIN[,1]))

sex<-as.data.frame(as.matrix(sexIN[newrowindex,1]))

age<-as.data.frame(as.matrix(ageIN[newrowindex,1]))

bmi<-as.data.frame(as.matrix(bmiIN[newrowindex,1]))

edyears<-as.data.frame(as.matrix(edyearsIN[newrowindex,1]))

Outputadmix<-NULL

OutputGWAS<-NULL

Outputjoint<-NULL

#################

#Each simulated site is now tested for association.

#################

for (n in 1:ncol(headers)){

admixture_posterior<-NULL

GWAS_posterior<-NULL

joint_posterior<-NULL

localanc<-c(localanc1[[n]])

geno<-c(geno1[[n]])

y<-c(phenotype)

globalanc<-c(GlobalMeanAncestry)

posterior <- function(x,prior,lambda)

{(dchisq(x,1,lambda)*prior)/((dchisq(x,1,lambda)*prior)+(dchisq(x,1,0)*(1-prior)))}

admixture_burden <- 78.82 #for the Tallahassee data

association_burden <- 27559 #for the Tallahassee data

#################

#In the following section the admixture posterior is calculated using both #local and global ancestry while taking into account the number of tests.

#The admixture posterior will later be used as a prior for the joint. #association test.

#################

result <-

summary(glm(y$V1~localanc+globalanc$V1+sex$V1+age$V1+bmi$V1+edyears$V1+pc1$V1+pc2$V1+pc3$V1+pc4$V1+pc5$V1+pc6$V1+pc7$V1+pc8$V1+pc9$V1+pc10$V1,family=gaussian))

admixture_p <- result$coefficients[2,4]

admixture_lambda <- (qnorm(1-0.05/admixture_burden/2)+qnorm(0.8))^2

admixture_prior <- 1/admixture_burden

admixture_test <- qchisq(admixture_p,1,0,lower.tail=FALSE)

admixture_posterior <-posterior(x=admixture_test,prior=admixture_prior,lambda=admixture_lambda)

#################

#Stratified association testing is performed where individual are grouped #based on local ancestry at that site.

#################

group0<-which(localanc==0)

if ((is.element(0,geno[group0]) & is.element(1,geno[group0])) |

(is.element(1,geno[group0]) & is.element(2,geno[group0])) |

(is.element(0,geno[group0]) & is.element(2,geno[group0])))

{

result <-

summary(glm(y$V1[group0]~geno[group0]+globalanc$V1[group0]+sex$V1[group0]+age$V1[group0]+edyears$V1[group0]+bmi$V1[group0]+pc1$V1[group0]+pc2$V1[group0]+pc3$V1[group0]+pc4$V1[group0]+pc5$V1[group0]+pc6$V1[group0]+pc7$V1[group0]+pc8$V1[group0]+pc9$V1[group0]+pc10$V1[group0],family=gaussian))

if (nrow(result$coefficients)==17){(tmp1 <-result$coefficients[2,1]) & (tmp2 <- result$coefficients[2,2])

}else{

(tmp1<-NA) & (tmp2<-NA)}

} else {

tmp1 <- NA

tmp2 <- NA

}

group1 <- which(localanc==1)

if ((is.element(0,geno[group1]) & is.element(1,geno[group1])) |

(is.element(1,geno[group1]) & is.element(2,geno[group1])) |

(is.element(0,geno[group1]) & is.element(2,geno[group1]))) {

result <-summary(glm(y$V1[group1]~geno[group1]+globalanc$V1[group1]+sex$V1[group1]+age$V1[group1]+edyears$V1[group1]+bmi$V1[group1]+pc1$V1[group1]+pc2$V1[group1]+pc3$V1[group1]+pc4$V1[group1]+pc5$V1[group1]+pc6$V1[group1]+pc7$V1[group1]+pc8$V1[group1]+pc9$V1[group1]+pc10$V1[group1],family=gaussian))

if (nrow(result$coefficients)==17){(tmp3 <-

result$coefficients[2,1]) & (tmp4 <- result$coefficients[2,2])

}else{

(tmp3<-NA) & (tmp4<-NA)}

} else {

tmp3 <- NA

tmp4 <- NA

}

group2 <- which(localanc==2)

if ((is.element(0,geno[group2]) & is.element(1,geno[group2])) |

(is.element(1,geno[group2]) & is.element(2,geno[group2])) |

(is.element(0,geno[group2]) & is.element(2,geno[group2]))) {

result <-

summary(glm(y$V1[group2]~geno[group2]+globalanc$V1[group2]+sex$V1[group2]+age$V1[group2]+edyears$V1[group2]+bmi$V1[group2]+pc1$V1[group2]+pc2$V1[group2]+pc3$V1[group2]+pc4$V1[group2]+pc5$V1[group2]+pc6$V1[group2]+pc7$V1[group2]+pc8$V1[group2]+pc9$V1[group2]+pc10$V1[group2],family=gaussian))

if (nrow(result$coefficients)==17){(tmp5 <-

result$coefficients[2,1]) & (tmp6 <- result$coefficients[2,2])

}else{

(tmp5<-NA) & (tmp6<-NA)}

} else {

tmp5 <- NA

tmp6 <- NA

}

#################

#Inverse variance-weighted fixed effects are then corrected for and the #p-values are pooled.

#################

pooled.pval <- NA

if (!any(is.na(c(tmp1,tmp2,tmp3,tmp4,tmp5,tmp6)))) {

pooled.beta <-

((tmp1/(tmp2^2))+(tmp3/(tmp4^2))+(tmp5/(tmp6^2)))/(1/(tmp2^2)+1/(tmp4^2)+1/(tmp6^2))

pooled.se <- sqrt(1/(1/(tmp2^2)+1/(tmp4^2)+1/(tmp6^2)))

pooled.pval <- 2*(1-pnorm(abs(pooled.beta/pooled.se)))

}

if (!is.na(tmp1) && !is.na(tmp2) && !is.na(tmp3) && !is.na(tmp4) &&

is.na(tmp5) && is.na(tmp6)) {

pooled.beta <-((tmp1/(tmp2^2))+(tmp3/(tmp4^2)))/(1/(tmp2^2)+1/(tmp4^2))

pooled.se <- sqrt(1/(1/(tmp2^2)+1/(tmp4^2)))

pooled.pval <- 2*(1-pnorm(abs(pooled.beta/pooled.se)))

}

if (!is.na(tmp1) && !is.na(tmp2) && is.na(tmp3) && is.na(tmp4) &&

!is.na(tmp5) && !is.na(tmp6)) {

pooled.beta <-((tmp1/(tmp2^2))+(tmp5/(tmp6^2)))/(1/(tmp2^2)+1/(tmp6^2))

pooled.se <- sqrt(1/(1/(tmp2^2)+1/(tmp6^2)))

pooled.pval <- 2*(1-pnorm(abs(pooled.beta/pooled.se)))

}

if (is.na(tmp1) && is.na(tmp2) && !is.na(tmp3) && !is.na(tmp4) &&

!is.na(tmp5) && !is.na(tmp6)) {

pooled.beta <-((tmp3/(tmp4^2))+(tmp5/(tmp6^2)))/(1/(tmp4^2)+1/(tmp6^2))

pooled.se <- sqrt(1/(1/(tmp4^2)+1/(tmp6^2)))

pooled.pval <- 2*(1-pnorm(abs(pooled.beta/pooled.se)))

}

if (!is.na(tmp1) && !is.na(tmp2) && is.na(tmp3) && is.na(tmp4) &&

is.na(tmp5) && is.na(tmp6)) {

pooled.beta <- tmp1

pooled.se <- tmp2

pooled.pval <- 2*(1-pnorm(abs(pooled.beta/pooled.se)))

}

if (is.na(tmp1) && is.na(tmp2) && !is.na(tmp3) && !is.na(tmp4) &&

is.na(tmp5) && is.na(tmp6)) {

pooled.beta <- tmp3

pooled.se <- tmp4

pooled.pval <- 2*(1-pnorm(abs(pooled.beta/pooled.se)))

}

if (is.na(tmp1) && is.na(tmp2) && is.na(tmp3) && is.na(tmp4) &&

!is.na(tmp5) && !is.na(tmp6)) {

pooled.beta <- tmp5

pooled.se <- tmp6

pooled.pval <- 2*(1-pnorm(abs(pooled.beta/pooled.se)))

}

#################

#The joint posterior is now generated based on the pooled p-value with the #admixture posterior being used as a prior for the joint test.

#################

if (!is.na(pooled.pval))

{

association_lambda <- (qnorm(1-

0.05/association_burden/2)+qnorm(0.8))^2

association_test <- qchisq(pooled.pval,1,0,lower.tail=FALSE)

joint_posterior <- posterior(x=association_test, prior=admixture_posterior, lambda=association_lambda)

} else {

joint_posterior <- NA

}

association_prior <- 1/association_burden

GWAS_posterior<- posterior(x=association_test, prior=association_prior, lambda=association_lambda)

Outputadmix[n]<-admixture_posterior

OutputGWAS[n]<-GWAS_posterior

Outputjoint[n]<-joint_posterior

}

#################

#The output of each simulation is then saved.

#################

simoutputadmix[,k]<-Outputadmix

simoutputGWAS[,k]<-Outputadmix

simoutputjoint[,k]<-Outputadmix

}

simoutput<-list(simoutputadmix, simoutputGWAS, simoutputjoint)

simoutput

}

#################

#Running the function:

#For computational efficiency, the simulations were split across as number #of different processors using the doParallel and foreach packages.

#doParallel detects the number of processors in the systems and registers #them for use by the foreach function.

#################

library(doParallel)

ncore<-detectCores()

cl<-makeCluster(as.numeric(ncore))

registerDoParallel(cl)

library(foreach)

combinelist<- function(oldlist,newlist) {

out<-oldlist

for(n in 1:length(oldlist)) {

out[[n]]<-cbind(oldlist[[n]],newlist[[n]])

}

out

}

#################

#Covariate and phenotype data is read in:

#phenotype is the vector of phenotypes.

#localanc is the vector of local ancestry estimates at a marker.

#globalanc is the vector of global ancestry estimates at a marker.

#geno is the vector of recoded genotypes at a marker.

#################

phenotypeSBPIN<-read.table(file="157_sbp10.tsv", header=F,sep="\t", na.strings="NA")

phenotypeDBPIN<-read.table(file="157_dbp2.tsv", header=F,sep="\t", na.strings="NA")

GlobalMeanAncestryIN<-read.table(file="157_Global_AA_27559.tsv", header=F,sep="\t", na.strings="NA")

sexIN<-read.table(file="157_sex.tsv", header=F,sep="\t", na.strings="NA")

ageIN<-read.table(file="157_age.tsv", header=F,sep="\t", na.strings="NA")

bmiIN<-read.table(file="157_bmi.tsv", header=F,sep="\t", na.strings="NA")

edyearsIN<-read.table(file="157_edyears.tsv", header=F,sep="\t", na.strings="NA")

PCair1<-read.table(file="157_pcair1.tsv", header=F,sep="\t", na.strings="NA")

PCair2<-read.table(file="157_pcair2.tsv", header=F,sep="\t", na.strings="NA")

PCair3<-read.table(file="157_pcair3.tsv", header=F,sep="\t", na.strings="NA")

PCair4<-read.table(file="157_pcair4.tsv", header=F,sep="\t", na.strings="NA")

PCair5<-read.table(file="157_pcair5.tsv", header=F,sep="\t", na.strings="NA")

PCair6<-read.table(file="157_pcair6.tsv", header=F,sep="\t", na.strings="NA")

PCair7<-read.table(file="157_pcair7.tsv", header=F,sep="\t", na.strings="NA")

PCair8<-read.table(file="157_pcair8.tsv", header=F,sep="\t", na.strings="NA")

PCair9<-read.table(file="157_pcair9.tsv", header=F,sep="\t", na.strings="NA")

PCair10<-read.table(file="157_pcair10.tsv", header=F,sep="\t", na.strings="NA")

#################

#Example Model 1 SBP BMIX

#################

set.seed(385)

localanc1<-read.table(file="Model1_SBP_Anc.tsv", header=F,sep="\t", na.strings="NA")

geno1<-read.table(file="Model1_SBP_Geno.tsv",header=F,sep="\t", na.strings="NA")

headers<-read.csv(file="Model1_SBP_Header.csv",sep=",")

#################

#Note: The number of simulations stated in the foreach command reflects #the number handled by each processor, since 8 processors were used the #total is 10,000 simulations (8*1250=10,000)

#################

finaloutputedyears<-foreach(times(8), .combine = combinelist) %dopar% BMIXsimed(localanc1=localanc1, geno1=geno1, phenotypeIN=phenotypeSBPIN, GlobalMeanAncestryIN=GlobalMeanAncestryIN, sexIN=sexIN, ageIN=ageIN, bmiIN=bmiIN, edyearsIN=edyearsIN, PCair1=PCair1, PCair2=PCair2, PCair3 = PCair3, PCair4 = PCair4, PCair5 = PCair5, PCair6 = PCair6, PCair7 = PCair7, PCair8 = PCair8, PCair9 = PCair9, PCair10 = PCair10, headers = headers, n.sims=1250)

write.csv(t(finaloutputedyears[[1]]), file="BMIXSig_Resample_Admix_Model1_edyears_BP10_Output_SBP.csv")

write.csv(t(finaloutputedyears[[2]]), file="BMIXSig_Resample_GWAS_Model1_edyears_BP10_Output_SBP.csv")

write.csv(t(finaloutputedyears[[3]]), file="BMIXSig_Resample_Joint_Model1_edyears_BP10_Output_SBP.csv")

rm(finaloutputedyears)

#################
#################

#Simulation ancestry and association analysis Model 2

#Implementation of the BMIX test

#adapted from Daniel Shriner, July 28,2011 by Jacklyn Quinlan, et al.

#Sept, 2015 and above association code

#################

#################

BMIXM2edsim<-function(localanc1, geno1, phenotypeIN, GlobalMeanAncestryIN, sexIN, ageIN, bmiIN, edyearsIN, utIN, PCair1, PCair2, PCair3, PCair4, PCair5, PCair6, PCair7, PCair8, PCair9, PCair10, headers, n.sims) {

rownumber<-1:157

pc1<-PCair1

pc2<-PCair2

pc3<-PCair3

pc4<-PCair4

pc5<-PCair5

pc6<-PCair6

pc7<-PCair7

pc8<-PCair8

pc9<-PCair9

pc10<-PCair10

#################

#Output matrices are defined by the number of sites tested and #simulations run.

#################

simoutputadmix<-matrix(nrow=ncol(headers),ncol=n.sims)

simoutputGWAS<-matrix(nrow=ncol(headers),ncol=n.sims)

simoutputjoint<-matrix(nrow=ncol(headers),ncol=n.sims)

simoutput<-list(simoutputadmix, simoutputGWAS, simoutputjoint)

for (k in 1:n.sims){

#################

#Row numbers are shuffled each run of the simulation

#################

newrowindex<-sample(rownumber)

#################

#Ancestry and relatedness remains linked to genotype, while phenotype, #sex, age, bmi, ut, and edyears are shuffled based on row numbers

#################

phenotype<-as.data.frame(as.matrix(phenotypeIN[newrowindex,1]))

GlobalMeanAncestry<-as.data.frame(as.matrix(GlobalMeanAncestryIN[,1]))

sex<-as.data.frame(as.matrix(sexIN[newrowindex,1]))

age<-as.data.frame(as.matrix(ageIN[newrowindex,1]))

bmi<-as.data.frame(as.matrix(bmiIN[newrowindex,1]))

ut<-as.data.frame(as.matrix(utIN[newrowindex,1]))

edyears<-as.data.frame(as.matrix(edyearsIN[newrowindex,1]))

Outputadmix<-NULL

OutputGWAS<-NULL

Outputjoint<-NULL

for (n in 1:ncol(headers)){

admixture_posterior<-NULL

GWAS_posterior<-NULL

joint_posterior<-NULL

localanc<-c(localanc1[[n]])

geno<-c(geno1[[n]])

utother<-c(ut)

y<-c(phenotype)

globalanc<-c(GlobalMeanAncestry)

posterior <- function(x,prior,lambda)

{(dchisq(x,1,lambda)*prior)/((dchisq(x,1,lambda)*prior)+(dchisq(x,1,0)*(1

-prior)))}

admixture_burden <- 78.82 #for the Tallahassee data

association_burden <- 27559 #for the Tallahassee data

#################

#In the following section the admixture posterior is calculated using both #local and global ancestry while taking into account the number of tests.

#The admixture posterior will later be used as a prior for the joint. #association test.

#################

result <-summary(glm(y$V1~localanc+globalanc$V1+utother$V1+sex$V1+age$V1+bmi$V1+edyears$V1+pc1$V1+pc2$V1+pc3$V1+pc4$V1+pc5$V1+pc6$V1+pc7$V1+pc8$V1+pc9$V1+pc10$V1,family=gaussian))

admixture_p <- result$coefficients[2,4]

admixture_lambda <- (qnorm(1-0.05/admixture_burden/2)+qnorm(0.8))^2

admixture_prior <- 1/admixture_burden

admixture_test <- qchisq(admixture_p,1,0,lower.tail=FALSE)

admixture_posterior <-

posterior(x=admixture_test,prior=admixture_prior,lambda=admixture_lambda)

#################

#Stratified association testing is performed where individual are grouped #based on local ancestry at that site.

#################

group0<-which(localanc==0)

if ((is.element(0,geno[group0]) & is.element(1,geno[group0])) |

(is.element(1,geno[group0]) & is.element(2,geno[group0])) |

(is.element(0,geno[group0]) & is.element(2,geno[group0])))

{

result <-summary(glm(y$V1[group0]~geno[group0]+globalanc$V1[group0]+utother$V1[group0]+sex$V1[group0]+age$V1[group0]+bmi$V1[group0]+edyears$V1[group0]+pc1$V1[group0]+pc2$V1[group0]+pc3$V1[group0]+pc4$V1[group0]+pc5$V1[group0]+pc6$V1[group0]+pc7$V1[group0]+pc8$V1[group0]+pc9$V1[group0]+pc10$V1[group0],family=gaussian))

if (nrow(result$coefficients)==18){(tmp1 <-result$coefficients[2,1]) & (tmp2 <- result$coefficients[2,2])

}else{

(tmp1<-NA) & (tmp2<-NA)}

} else {

tmp1 <- NA

tmp2 <- NA

}

group1 <- which(localanc==1)

if ((is.element(0,geno[group1]) & is.element(1,geno[group1])) |

(is.element(1,geno[group1]) & is.element(2,geno[group1])) |

(is.element(0,geno[group1]) & is.element(2,geno[group1]))) {

result <-summary(glm(y$V1[group1]~geno[group1]+globalanc$V1[group1]+utother$V1[group1]+sex$V1[group1]+age$V1[group1]+bmi$V1[group1]+edyears$V1[group1]+pc1$V1[group1]+pc2$V1[group1]+pc3$V1[group1]+pc4$V1[group1]+pc5$V1[group1]+pc6$V1[group1]+pc7$V1[group1]+pc8$V1[group1]+pc9$V1[group1]+pc10$V1[group1],family=gaussian))

if (nrow(result$coefficients)==18){(tmp3 <-result$coefficients[2,1]) & (tmp4 <- result$coefficients[2,2])

}else{

(tmp3<-NA) & (tmp4<-NA)}

} else {

tmp3 <- NA

tmp4 <- NA

}

group2 <- which(localanc==2)

if ((is.element(0,geno[group2]) & is.element(1,geno[group2])) |

(is.element(1,geno[group2]) & is.element(2,geno[group2])) |

(is.element(0,geno[group2]) & is.element(2,geno[group2]))) {

result <-summary(glm(y$V1[group2]~geno[group2]+globalanc$V1[group2]+utother$V1[group2]+sex$V1[group2]+age$V1[group2]+bmi$V1[group2]+edyears$V1[group2]+pc1$V1[group2]+pc2$V1[group2]+pc3$V1[group2]+pc4$V1[group2]+pc5$V1[group2]+pc6$V1[group2]+pc7$V1[group2]+pc8$V1[group2]+pc9$V1[group2]+pc10$V1[group2],family=gaussian))

if (nrow(result$coefficients)==18){(tmp5 <-result$coefficients[2,1]) & (tmp6 <- result$coefficients[2,2])

}else{

(tmp5<-NA) & (tmp6<-NA)}

} else {

tmp5 <- NA

tmp6 <- NA

}

#################

#Inverse variance-weighted fixed effects are then corrected for and the #p-values are pooled.

#################

pooled.pval <- NA

if (!any(is.na(c(tmp1,tmp2,tmp3,tmp4,tmp5,tmp6)))) {

pooled.beta <-

((tmp1/(tmp2^2))+(tmp3/(tmp4^2))+(tmp5/(tmp6^2)))/(1/(tmp2^2)+1/(tmp4^2)+

1/(tmp6^2))

pooled.se <- sqrt(1/(1/(tmp2^2)+1/(tmp4^2)+1/(tmp6^2)))

pooled.pval <- 2*(1-pnorm(abs(pooled.beta/pooled.se)))

}

if (!is.na(tmp1) && !is.na(tmp2) && !is.na(tmp3) && !is.na(tmp4) &&

is.na(tmp5) && is.na(tmp6)) {

pooled.beta <-

((tmp1/(tmp2^2))+(tmp3/(tmp4^2)))/(1/(tmp2^2)+1/(tmp4^2))

pooled.se <- sqrt(1/(1/(tmp2^2)+1/(tmp4^2)))

pooled.pval <- 2*(1-pnorm(abs(pooled.beta/pooled.se)))

}

if (!is.na(tmp1) && !is.na(tmp2) && is.na(tmp3) && is.na(tmp4) &&

!is.na(tmp5) && !is.na(tmp6)) {

pooled.beta <-

((tmp1/(tmp2^2))+(tmp5/(tmp6^2)))/(1/(tmp2^2)+1/(tmp6^2))

pooled.se <- sqrt(1/(1/(tmp2^2)+1/(tmp6^2)))

pooled.pval <- 2*(1-pnorm(abs(pooled.beta/pooled.se)))

}

if (is.na(tmp1) && is.na(tmp2) && !is.na(tmp3) && !is.na(tmp4) &&

!is.na(tmp5) && !is.na(tmp6)) {

pooled.beta <-

((tmp3/(tmp4^2))+(tmp5/(tmp6^2)))/(1/(tmp4^2)+1/(tmp6^2))

pooled.se <- sqrt(1/(1/(tmp4^2)+1/(tmp6^2)))

pooled.pval <- 2*(1-pnorm(abs(pooled.beta/pooled.se)))

}

if (!is.na(tmp1) && !is.na(tmp2) && is.na(tmp3) && is.na(tmp4) &&

is.na(tmp5) && is.na(tmp6)) {

pooled.beta <- tmp1

pooled.se <- tmp2

pooled.pval <- 2*(1-pnorm(abs(pooled.beta/pooled.se)))

}

if (is.na(tmp1) && is.na(tmp2) && !is.na(tmp3) && !is.na(tmp4) &&

is.na(tmp5) && is.na(tmp6)) {

pooled.beta <- tmp3

pooled.se <- tmp4

pooled.pval <- 2*(1-pnorm(abs(pooled.beta/pooled.se)))

}

if (is.na(tmp1) && is.na(tmp2) && is.na(tmp3) && is.na(tmp4) &&

!is.na(tmp5) && !is.na(tmp6)) {

pooled.beta <- tmp5

pooled.se <- tmp6

pooled.pval <- 2*(1-pnorm(abs(pooled.beta/pooled.se)))

}

#################

#The joint posterior is now generated based on the pooled p-value with the #admixture posterior being used as a prior for the joint test.

#################

if (!is.na(pooled.pval))

{

association_lambda <- (qnorm(1-

0.05/association_burden/2)+qnorm(0.8))^2

association_test <- qchisq(pooled.pval,1,0,lower.tail=FALSE)

joint_posterior <- posterior(x=association_test,

prior=admixture_posterior, lambda=association_lambda)

} else {

joint_posterior <- NA

}

association_prior <- 1/association_burden

GWAS_posterior<- posterior(x=association_test, prior=association_prior,

lambda=association_lambda)

Outputadmix[n]<-admixture_posterior

OutputGWAS[n]<-GWAS_posterior

Outputjoint[n]<-joint_posterior

}

#################

#The output of each simulation is saved

#################

simoutput[[1]][,k]<-Outputadmix

simoutput[[2]][,k]<-OutputGWAS

simoutput[[3]][,k]<-Outputjoint

}

simoutput

}

#################

#Running the function:

#For computational efficiency, the simulations were split across as number #of different processors using the doParallel and foreach packages.

#doParallel detects the number of processors in the systems and registers #them for use by the foreach function.

#################

library(doParallel)

ncore<-detectCores()

cl<-makeCluster(as.numeric(ncore))

registerDoParallel(cl)

library(foreach)

combinelist<- function(oldlist,newlist) {

out<-oldlist

for(n in 1:length(oldlist)) {

out[[n]]<-cbind(oldlist[[n]],newlist[[n]])

}

out

}

#################

#Covariate and phenotype data is read in:

#phenotype is the vector of phenotypes.

#localanc is the vector of local ancestry estimates at a marker.

#globalanc is the vector of global ancestry estimates at a marker.

#geno is the vector of recoded genotypes at a marker.

#################

phenotypeSBPIN<-read.table(file="157_sbp10.tsv", header=F,sep="\t", na.strings="NA")

phenotypeDBPIN<-read.table(file="157_dbp2.tsv", header=F,sep="\t", na.strings="NA")

GlobalMeanAncestryIN<-read.table(file="157_Global_AA_27559.tsv", header=F,sep="\t", na.strings="NA")

sexIN<-read.table(file="157_sex.tsv", header=F,sep="\t", na.strings="NA")

ageIN<-read.table(file="157_age.tsv", header=F,sep="\t", na.strings="NA")

bmiIN<-read.table(file="157_bmi.tsv", header=F,sep="\t", na.strings="NA")

utotherIN<-read.table(file="157_utother.tsv", header=F,sep="\t", na.strings="NA")

edyearsIN<-read.table(file="157_edyears.tsv", header=F,sep="\t", na.strings="NA")

PCair1<-read.table(file="157_pcair1.tsv", header=F,sep="\t", na.strings="NA")

PCair2<-read.table(file="157_pcair2.tsv", header=F,sep="\t", na.strings="NA")

PCair3<-read.table(file="157_pcair3.tsv", header=F,sep="\t", na.strings="NA")

PCair4<-read.table(file="157_pcair4.tsv", header=F,sep="\t", na.strings="NA")

PCair5<-read.table(file="157_pcair5.tsv", header=F,sep="\t", na.strings="NA")

PCair6<-read.table(file="157_pcair6.tsv", header=F,sep="\t", na.strings="NA")

PCair7<-read.table(file="157_pcair7.tsv", header=F,sep="\t", na.strings="NA")

PCair8<-read.table(file="157_pcair8.tsv", header=F,sep="\t", na.strings="NA")

PCair9<-read.table(file="157_pcair9.tsv", header=F,sep="\t", na.strings="NA")

PCair10<-read.table(file="157_pcair10.tsv", header=F,sep="\t", na.strings="NA")

#################

#Example Model 2 UTother SBP BMIX

#################

set.seed(385)

localanc1<-read.table(file="Model2_SBP_UTother_Anc.tsv", header=F,sep="\t", na.strings="NA")

geno1<-read.table(file="Model2_SBP_UTother_Geno.tsv",header=F,sep="\t", na.strings="NA")

headers<-read.csv(file="Model2_SBP_UTother_Header.csv",sep=",")

#################

#Note: The number of simulations stated in the foreach command reflects #the number handled by each processor, since 8 processors were used the #total is 10,000 simulations (8*1250=10,000)

#################

finaloutputedyears<-foreach(times(8), .combine = combinelist) %dopar% BMIXM2edsim(localanc1, geno1, phenotypeSBPIN, GlobalMeanAncestryIN, sexIN, ageIN, bmiIN, edyearsIN, utotherIN, PCair1, PCair2, PCair3, PCair4, PCair5, PCair6, PCair7, PCair8, PCair9, PCair10, headers, n.sims=1250)

write.csv(t(finaloutputedyears[[1]]), file="BMIXSig_Resample_Admix_Model2_UTother_edyears_BP10_Output_SBP.csv")

write.csv(t(finaloutputedyears[[2]]), file="BMIXSig_Resample_GWAS_Model2_UTother_edyears_BP10_Output_SBP.csv")

write.csv(t(finaloutputedyears[[3]]), file="BMIXSig_Resample_Joint_Model2_UTother_edyears_BP10_Output_SBP.csv")

rm(finaloutputedyears)

#################

#################

#Simulation ancestry and association analysis Model 3

#Implementation of the BMIX test

#adapted from Daniel Shriner, July 28,2011 by Jacklyn Quinlan, et al.

#Sept, 2015 and above association code

#################

#################

BMIXM3edsim<-function(localanc1, geno1, phenotypeIN, GlobalMeanAncestryIN, sexIN, ageIN, bmiIN, edyearsIN, utIN, PCair1, PCair2, PCair3, PCair4, PCair5, PCair6, PCair7, PCair8, PCair9, PCair10, headers, n.sims) {

rownumber<-1:157

pc1<-PCair1

pc2<-PCair2

pc3<-PCair3

pc4<-PCair4

pc5<-PCair5

pc6<-PCair6

pc7<-PCair7

pc8<-PCair8

pc9<-PCair9

pc10<-PCair10

#################

#Output matrices are defined by the number of sites tested and #simulations run.

#################

simoutputadmix<-matrix(nrow=ncol(headers),ncol=n.sims)

simoutputGWAS<-matrix(nrow=ncol(headers),ncol=n.sims)

simoutputjoint<-matrix(nrow=ncol(headers),ncol=n.sims)

simoutput<-list(simoutputadmix, simoutputGWAS, simoutputjoint)

for (k in 1:n.sims){

#################

#Row numbers are shuffled each run of the simulation.

#################

newrowindex<-sample(rownumber)

#################

#Ancestry and relatedness remains linked to genotype, while phenotype, #sex, age, bmi, ut, and edyears are shuffled based on row numbers

#################

phenotype<-as.data.frame(as.matrix(phenotypeIN[newrowindex,1]))

GlobalMeanAncestry<-as.data.frame(as.matrix(GlobalMeanAncestryIN[,1]))

sex<-as.data.frame(as.matrix(sexIN[newrowindex,1]))

age<-as.data.frame(as.matrix(ageIN[newrowindex,1]))

bmi<-as.data.frame(as.matrix(bmiIN[newrowindex,1]))

ut<-as.data.frame(as.matrix(utIN[newrowindex,1]))

edyears<-as.data.frame(as.matrix(edyearsIN[newrowindex,1]))

Outputadmix<-NULL

OutputGWAS<-NULL

Outputjoint<-NULL

#################

#Each simulated site is now tested for association.

#################

for (n in 1:ncol(headers)){

admixture_posterior<-NULL

GWAS_posterior<-NULL

joint_posterior<-NULL

localanc<-c(localanc1[[n]])

geno<-c(geno1[[n]])

utother<-c(ut)

y<-c(phenotype)

globalanc<-c(GlobalMeanAncestry)

posterior <- function(x,prior,lambda)

{(dchisq(x,1,lambda)*prior)/((dchisq(x,1,lambda)*prior)+(dchisq(x,1,0)*(1

-prior)))}

admixture_burden <- 78.82166 #for the Tallahassee data

association_burden <- 18324 #for MAF10% out of the Tallahassee 27559 SNPs

#################

#In the following section the admixture posterior is calculated using both #local and global ancestry while taking into account the number of tests.

#The admixture posterior will later be used as a prior for the joint. #association test.

#################

result <-summary(glm(y$V1~localanc+globalanc$V1+utother$V1+sex$V1+age$V1+bmi$V1+edyears$V1+utother$V1*localanc+pc1$V1+pc2$V1+pc3$V1+pc4$V1+pc5$V1+pc6$V1+pc7$V1+pc8$V1+pc9$V1+pc10$V1,family=gaussian))

admixture_p <- result$coefficients[19,4]

admixture_lambda <- (qnorm(1-0.05/admixture_burden/2)+qnorm(0.8))^2

admixture_prior <- 1/admixture_burden

admixture_test <- qchisq(admixture_p,1,0,lower.tail=FALSE)

admixture_posterior <-

posterior(x=admixture_test,prior=admixture_prior,lambda=admixture_lambda)

#################

#Stratified association testing is performed where individual are grouped #based on local ancestry at that site.

#################

group0<-which(localanc==0)

if ((is.element(0,geno[group0]) & is.element(1,geno[group0])) |

(is.element(1,geno[group0]) & is.element(2,geno[group0])) |

(is.element(0,geno[group0]) & is.element(2,geno[group0])))

{

result <-summary(glm(y$V1[group0]~geno[group0]+globalanc$V1[group0]+utother$V1[group0]+sex$V1[group0]+age$V1[group0]+bmi$V1[group0]+edyears$V1[group0]+utother$V1[group0]*geno[group0]+pc1$V1[group0]+pc2$V1[group0]+pc3$V1[group0]+pc4$V1[group0]+pc5$V1[group0]+pc6$V1[group0]+pc7$V1[group0]+pc8$V1[group0]+pc9$V1[group0]+pc10$V1[group0],family=gaussian))

if (nrow(result$coefficients)==19){(tmp1 <-result$coefficients[19,1]) & (tmp2 <- result$coefficients[19,2])

}else{

(tmp1<-NA) & (tmp2<-NA)}

} else {

tmp1 <- NA

tmp2 <- NA

}

group1 <- which(localanc==1)

if ((is.element(0,geno[group1]) & is.element(1,geno[group1])) |

(is.element(1,geno[group1]) & is.element(2,geno[group1])) |

(is.element(0,geno[group1]) & is.element(2,geno[group1]))) {

result <-summary(glm(y$V1[group1]~geno[group1]+globalanc$V1[group1]+utother$V1[group1]+sex$V1[group1]+age$V1[group1]+bmi$V1[group1]+edyears$V1[group1]+ utother$V1[group1]*geno[group1]+pc1$V1[group1]+pc2$V1[group1]+pc3$V1[group1]+pc4$V1[group1]+pc5$V1[group1]+pc6$V1[group1]+pc7$V1[group1]+pc8$V1[group1]+pc9$V1[group1]+pc10$V1[group1],family=gaussian))

if (nrow(result$coefficients)==19){(tmp3 <-result$coefficients[19,1]) & (tmp4 <- result$coefficients[19,2])

}else{

(tmp3<-NA) & (tmp4<-NA)}

} else {

tmp3 <- NA

tmp4 <- NA

}

group2 <- which(localanc==2)

if ((is.element(0,geno[group2]) & is.element(1,geno[group2])) |

(is.element(1,geno[group2]) & is.element(2,geno[group2])) |

(is.element(0,geno[group2]) & is.element(2,geno[group2]))) {

result <-summary(glm(y$V1[group2]~geno[group2]+globalanc$V1[group2]+ utother$V1[group2]+sex$V1[group2]+age$V1[group2]+bmi$V1[group2]+edyears$V1[group2]+utother$V1[group2]*geno[group2]+pc1$V1[group2]+pc2$V1[group2]+pc3$V1[group2]+pc4$V1[group2]+pc5$V1[group2]+pc6$V1[group2]+pc7$V1[group2]+pc8$V1[group2]+pc9$V1[group2]+pc10$V1[group2],family=gaussian))

if (nrow(result$coefficients)==19){(tmp5 <-result$coefficients[19,1]) & (tmp6 <- result$coefficients[19,2])

}else{

(tmp5<-NA) & (tmp6<-NA)}

} else {

tmp5 <- NA

tmp6 <- NA

}

#################

#Inverse variance-weighted fixed effects are then corrected for and the #p-values are pooled.

#################

pooled.pval <- NA

if (!any(is.na(c(tmp1,tmp2,tmp3,tmp4,tmp5,tmp6)))) {

pooled.beta <-((tmp1/(tmp2^2))+(tmp3/(tmp4^2))+(tmp5/(tmp6^2)))/(1/(tmp2^2)+1/(tmp4^2)+1/(tmp6^2))

pooled.se <- sqrt(1/(1/(tmp2^2)+1/(tmp4^2)+1/(tmp6^2)))

pooled.pval <- 2*(1-pnorm(abs(pooled.beta/pooled.se)))

}

if (!is.na(tmp1) && !is.na(tmp2) && !is.na(tmp3) && !is.na(tmp4) &&

is.na(tmp5) && is.na(tmp6)) {

pooled.beta <-

((tmp1/(tmp2^2))+(tmp3/(tmp4^2)))/(1/(tmp2^2)+1/(tmp4^2))

pooled.se <- sqrt(1/(1/(tmp2^2)+1/(tmp4^2)))

pooled.pval <- 2*(1-pnorm(abs(pooled.beta/pooled.se)))

}

if (!is.na(tmp1) && !is.na(tmp2) && is.na(tmp3) && is.na(tmp4) &&

!is.na(tmp5) && !is.na(tmp6)) {

pooled.beta <-((tmp1/(tmp2^2))+(tmp5/(tmp6^2)))/(1/(tmp2^2)+1/(tmp6^2))

pooled.se <- sqrt(1/(1/(tmp2^2)+1/(tmp6^2)))

pooled.pval <- 2*(1-pnorm(abs(pooled.beta/pooled.se)))

}

if (is.na(tmp1) && is.na(tmp2) && !is.na(tmp3) && !is.na(tmp4) &&

!is.na(tmp5) && !is.na(tmp6)) {

pooled.beta <-((tmp3/(tmp4^2))+(tmp5/(tmp6^2)))/(1/(tmp4^2)+1/(tmp6^2))

pooled.se <- sqrt(1/(1/(tmp4^2)+1/(tmp6^2)))

pooled.pval <- 2*(1-pnorm(abs(pooled.beta/pooled.se)))

}

if (!is.na(tmp1) && !is.na(tmp2) && is.na(tmp3) && is.na(tmp4) &&

is.na(tmp5) && is.na(tmp6)) {

pooled.beta <- tmp1

pooled.se <- tmp2

pooled.pval <- 2*(1-pnorm(abs(pooled.beta/pooled.se)))

}

if (is.na(tmp1) && is.na(tmp2) && !is.na(tmp3) && !is.na(tmp4) &&

is.na(tmp5) && is.na(tmp6)) {

pooled.beta <- tmp3

pooled.se <- tmp4

pooled.pval <- 2*(1-pnorm(abs(pooled.beta/pooled.se)))

}

if (is.na(tmp1) && is.na(tmp2) && is.na(tmp3) && is.na(tmp4) &&

!is.na(tmp5) && !is.na(tmp6)) {

pooled.beta <- tmp5

pooled.se <- tmp6

pooled.pval <- 2*(1-pnorm(abs(pooled.beta/pooled.se)))

}

#################

#The joint posterior is now generated based on the pooled p-value with the #admixture posterior being used as a prior for the joint test.

#################

if (!is.na(pooled.pval))

{

association_lambda <- (qnorm(1-

0.05/association_burden/2)+qnorm(0.8))^2

association_test <- qchisq(pooled.pval,1,0,lower.tail=FALSE)

joint_posterior <- posterior(x=association_test,

prior=admixture_posterior, lambda=association_lambda)

} else {

joint_posterior <- NA

}

association_prior <- 1/association_burden

GWAS_posterior<- posterior(x=association_test, prior=association_prior,lambda=association_lambda)

Outputadmix[n]<-admixture_posterior

OutputGWAS[n]<-GWAS_posterior

Outputjoint[n]<-joint_posterior

}

#################

#The output of each simulation is saved

#################

simoutput[[1]][,k]<-Outputadmix

simoutput[[2]][,k]<-OutputGWAS

simoutput[[3]][,k]<-Outputjoint

}

simoutput

}

#################

#Running the function:

#For computational efficiency, the simulations were split across as number #of different processors using the doParallel and foreach packages.

#doParallel detects the number of processors in the systems and registers #them for use by the foreach function.

#################

library(doParallel)

ncore<-detectCores()

cl<-makeCluster(as.numeric(ncore))

registerDoParallel(cl)

library(foreach)

combinelist<- function(oldlist,newlist) {

out<-oldlist

for(n in 1:length(oldlist)) {

out[[n]]<-cbind(oldlist[[n]],newlist[[n]])

}

out

}

#################

#Covariate and phenotype data is read in:

#phenotype is the vector of phenotypes.

#localanc is the vector of local ancestry estimates at a marker.

#globalanc is the vector of global ancestry estimates at a marker.

#geno is the vector of recoded genotypes at a marker.

#################

phenotypeSBPIN<-read.table(file="157_sbp10.tsv", header=F,sep="\t", na.strings="NA")

phenotypeDBPIN<-read.table(file="157_dbp2.tsv", header=F,sep="\t", na.strings="NA")

GlobalMeanAncestryIN<-read.table(file="157_Global_AA_27559.tsv", header=F,sep="\t", na.strings="NA")

sexIN<-read.table(file="157_sex.tsv", header=F,sep="\t", na.strings="NA")

ageIN<-read.table(file="157_age.tsv", header=F,sep="\t", na.strings="NA")

bmiIN<-read.table(file="157_bmi.tsv", header=F,sep="\t", na.strings="NA")

utotherIN<-read.table(file="157_ utother.tsv", header=F,sep="\t", na.strings="NA")

utcumIN<-read.table(file="157_utcumulative.tsv", header=F,sep="\t", na.strings="NA")

utedIN<-read.table(file="157_uted.tsv", header=F,sep="\t", na.strings="NA")

utvicIN<-read.table(file="157_utvicarious.tsv", header=F,sep="\t", na.strings="NA")

utotherIN<-read.table(file="157_utother.tsv", header=F,sep="\t", na.strings="NA")

utselfIN<-read.table(file="157_utself.tsv", header=F,sep="\t", na.strings="NA")

edyearsIN<-read.table(file="157_edyears.tsv", header=F,sep="\t", na.strings="NA")

PCair1<-read.table(file="157_pcair1.tsv", header=F,sep="\t", na.strings="NA")

PCair2<-read.table(file="157_pcair2.tsv", header=F,sep="\t", na.strings="NA")

PCair3<-read.table(file="157_pcair3.tsv", header=F,sep="\t", na.strings="NA")

PCair4<-read.table(file="157_pcair4.tsv", header=F,sep="\t", na.strings="NA")

PCair5<-read.table(file="157_pcair5.tsv", header=F,sep="\t", na.strings="NA")

PCair6<-read.table(file="157_pcair6.tsv", header=F,sep="\t", na.strings="NA")

PCair7<-read.table(file="157_pcair7.tsv", header=F,sep="\t", na.strings="NA")

PCair8<-read.table(file="157_pcair8.tsv", header=F,sep="\t", na.strings="NA")

PCair9<-read.table(file="157_pcair9.tsv", header=F,sep="\t", na.strings="NA")

PCair10<-read.table(file="157_pcair10.tsv", header=F,sep="\t", na.strings="NA")

#################

#Example Model 3 utother SBP BMIX

#################

set.seed(385)

localanc1<-read.table(file="Model3_SBP_UTother_Anc.tsv", header=F,sep="\t", na.strings="NA")

geno1<-read.table(file="Model3_SBP_UTother_Geno.tsv",header=F,sep="\t", na.strings="NA")

headers<-read.csv(file="Model3_SBP_UTother_Header.csv",sep=",")

finaloutputedyears<-foreach(times(8), .combine = combinelist) %dopar% BMIXM3edsim(localanc1, geno1, phenotypeSBPIN, GlobalMeanAncestryIN, sexIN, ageIN, bmiIN, edyearsIN, utotherIN, PCair1, PCair2, PCair3, PCair4, PCair5, PCair6, PCair7, PCair8, PCair9, PCair10, headers, n.sims=1250)

write.csv(t(finaloutputedyears[[1]]), file="BMIXSig_Resample_Admix_Model3_UTotherYN_edyears_BP10_Output_SBP.csv")

write.csv(t(finaloutputedyears[[2]]), file="BMIXSig_Resample_GWAS_Model3_UTotherYN_edyears_BP10_Output_SBP.csv")

write.csv(t(finaloutputedyears[[3]]), file="BMIXSig_Resample_Joint_Model3_UTotherYN_edyears_BP10_Output_SBP.csv")

rm(finaloutputedyears)
