# Supplementary material for: Genetic Loci and Novel Discrimination Measures Associated with Blood Pressure Variation in African Americans Living in Tallahassee
Source: PLoS One. 2016 Dec 21;11(12):e0167700. doi: 10.1371/journal.pone.0167700 (PMC5176163; doi:10.1371/journal.pone.0167700)
Supplement: S3 Table — (Expanded version of Table 3). (PDF) [file pone.0167700.s003.pdf]

Model	UT	SNP	Chr	Position (bp)	Type	Closest Gene	MAF	Pooled β	Pooled Pval	Joint posterior
**Model 1**	N/A	rs77804878	7	151847753	Intronic	MLL3	0.03822	-20.903482	4.8392E-12	0.99999715
		rs114805596	7	152039579	Intronic	MLL3	0.0414	-19.505664	6.7863E-11	0.99998149
		rs17173425	7	152094629	Intronic	MLL3	0.01911	-28.038339	1.2307E-09	0.99976968
**Model 2**	UT-Self	rs2597955	12	11261224	Intronic	PRH1-PRR4	0.3057	-11.083326	1.4941E-12	0.99999881
		rs2600370	12	11274876	Intronic	PRH1-PRR4	0.3013	-11.98956	4.4409E-16	1
		rs2600368	12	11277248	Intronic	PRH1-PRR4	0.3057	11.0833261	1.4941E-12	0.99999881
		rs2708349	12	11281904	Intronic	PRH1-PRR4	0.3057	11.0833261	1.4941E-12	0.99999881
		rs2600362	12	11282252	Intronic	PRH1-PRR4	0.3057	11.0833261	1.4941E-12	0.99999881
		rs2597921	12	11287911	Intronic	PRH1-PRR4	0.3057	11.0833261	1.4941E-12	0.99999881
		rs2416545	12	11321850	Intronic	PRH1-PRR4	0.3057	11.0833261	1.4941E-12	0.99999881
		rs2445762	15	51617708	Intronic	CYP19A1	0.4331	8.45127777	5.04621E-08	0.87408336
	UT-Other	rs1319603	1	215631500	Downstream / upstream	KCNK2/KCTD3	0.07643	16.3554502	1.43434E-09	0.98423096
**Model 3 No/Yes**	SNP*UT-Self	rs11190458	10	102053992	Intronic	PKD2L1	0.3885	-15.052098	4.66762E-05	0.53071068
**Model 3 Low/High**	SNP*UT-Other	rs34712049	15	51560064	Intronic	CYP19A1	0.2675	-51.437359	0	1
